# Growth Hormone-Releasing Hormone Antagonists Increase Radiosensitivity in Non-Small Cell Lung Cancer Cells

**DOI:** 10.3390/ijms26073267

**Published:** 2025-04-01

**Authors:** Iacopo Gesmundo, Francesca Pedrolli, Francesca Romana Giglioli, Florian Jazaj, Giuseppina Granato, Alessia Bertoldo, Federica Bistolfi, Vanesa Gregorc, Anna Sapino, Luisella Righi, Renzhi Cai, Wei Sha, Medhi Wangpaichitr, Mauro Papotti, Ezio Ghigo, Umberto Ricardi, Andrew V. Schally, Riccarda Granata

**Affiliations:** 1 Division of Endocrinology, Diabetes and Metabolism, 10126 Turin, Italy; iacopo.gesmundo@unito.it (I.G.); francesca.pedrolli@unito.it (F.P.); florian.jazaj@unito.it (F.J.); giuseppina.granato@unito.it (G.G.); alessia.bertoldo@unito.it (A.B.); federica.bistolfi@unito.it (F.B.); ezio.ghigo@unito.it (E.G.); 2 Department of Medical Sciences, University of Turin, 10126 Turin, Italy; anna.sapino@unito.it; 3 Medical Physics Unit, A.O.U. Città della Salute e Della Scienza, 10126 Turin, Italy; francescaromanagiglioli@gmail.com; 4 Candiolo Cancer Institute, Fondazione del Piemonte per l’Oncologia (FPO)-IRCCS, 10060 Candiolo, Italy; vanesa.gregorc@ircc.it; 5 Department of Oncology, Pathology Unit, University of Turin, San Luigi Gonzaga Hospital, 10043 Orbassano, Italy; luisella.righi@unito.it; 6 Endocrine, Polypeptide, and Cancer Institute, Veterans Affairs Medical Center, Miami, FL 33125, USA; xyzcai135@gmail.com (R.C.); mwangpaichitr@med.miam (M.W.); aschally@med.miami.edu (A.V.S.); 7 South Florida VA Foundation for Research and Education, Veterans Affairs Medical Center, Miami, FL 33125, USA; weisha17@gmail.com; 8 Department of Medicine, Divisions of Medical/Oncology and Endocrinology, and the Department of Pathology, Miller School of Medicine, University of Miami, Miami, FL 33136, USA; 9 Sylvester Comprehensive Cancer Center, Miller School of Medicine, University of Miami, Miami, FL 33136, USA; 10 Department of Oncology, Pathology Unit, University of Turin, and Città Della Salute e Della Scienza Hospital, 10126 Turin, Italy; mauro.papotti@unito.it; 11 Department of Oncology, University of Turin, 10126 Turin, Italy; umberto.ricardi@unito.it

**Keywords:** GHRH, GHRH antagonists, NSCLC, ionizing radiation, radiosensitivity

## Abstract

Growth hormone-releasing hormone (GHRH) antagonists exert antitumor functions in different experimental cancers. However, their role in combination with radiotherapy in non-small cell lung cancer (NSCLC) remains unknown. Therefore, we investigated the radiosensitizing effect of GHRH antagonists in NSCLC. A549 and H522 NSCLC cell lines were exposed to ionizing radiation (IR) and GHRH antagonists MIA-602 and MIA-690, either individually or in combination. Cell viability and proliferation were evaluated by MTT, BrdU, flow cytofluorimetry, and clonogenic assays; gene and protein expression, signaling pathways, and apoptosis were analyzed by real-time PCR, Western blot, annexin staining, and caspase-3 assay. GHRH antagonists showed antitumor effects alone and potentiated IR-induced inhibition of cell viability and proliferation. The combination of MIA-690 and IR decreased the expression of GHRH receptor, its oncogenic splice variant 1, and IGF1 mRNA levels. Additionally, cell cycle inhibitors and proapoptotic markers were upregulated, whereas cyclins, oncogenic *MYC*, and the antiapoptotic protein Bcl-2 were downregulated. Radioresistance was prevented by MIA-690, which also blunted epithelial–mesenchymal transition by enhancing E-cadherin and reducing mesenchymal, oxidative, and proangiogenic effectors. Finally, both MIA-602 and MIA-690 enhanced radiosensitivity in primary human NSCLC cells. These findings highlight the potential of GHRH antagonists as radiosensitizers in NSCLC treatment.

## 1. Introduction

Lung cancer is one of the most common neoplasia and a major health problem worldwide, with an estimated 1.8 million annual deaths, accounting for approximately 18% of global cancer mortality [1]. Histologically, lung cancer is categorized into small cell lung cancer (SCLC; ≈15% of cases) and non-small cell lung cancer (NSCLC; ≈85% of cases). NSCLC primarily includes lung adenocarcinoma (LUAD) and lung squamous cell carcinoma [2]. Although a proportion of NSCLC is non-smoking related, cigarette smoking is the predominant risk factor for the development of lung cancer, while other causes include genetic predisposition, lifestyle, environmental, and occupational exposures, and chronic lung disease [3]. Surgical resection is typically considered only for early-stage localized NSCLC, while radiotherapy with ionizing radiation (IR), either alone or in combination with chemotherapy or immunotherapy, remains the first-line treatment for advanced and metastatic tumors [4]. However, radiotherapy has several limitations, including radiation resistance, local recurrence, and metastasis. Notably, IR can enhance cancer cell migration and invasion by promoting hypoxia, epithelial–mesenchymal transition (EMT), and vascular damage, ultimately leading to poor prognosis [5]. Thus, new therapeutic strategies are essential to enhance radiotherapy effectiveness and mitigate its potential adverse effects.

The hypothalamic hormone growth hormone-releasing hormone (GHRH), apart from stimulating the release of pituitary growth hormone (GH), exerts various peripheral effects by binding to GHRH receptor (GHRH-R) and its splice variant 1 (SV1) [6,7]. In fact, GHRH and its agonistic analogs have been shown to possess neuroprotective, cardioprotective, anti-inflammatory, and antidiabetic properties [7,8,9,10,11]. Additionally, GHRH and its receptors have been identified in extrapituitary cells and tissues, where locally produced GHRH acts as an autocrine/paracrine growth factor [7,12]. The stimulatory loop formed by GHRH and its receptors can be blocked by GHRH antagonists, which exhibit antitumor functions across various cancer types, by directly inhibiting SV1 signaling or blocking tumor-derived insulin-like growth factor 1 (IGF1) and IGF2. Previous studies have shown that both early and latest series of GHRH antagonists, including those from the MIAMI (MIA) class, suppress cell proliferation and tumor growth in vitro and in vivo in experimental SCLC and NSCLC by hampering proliferative and survival pathways, promoting apoptosis and reactive oxygen species (ROS) production, and reducing the expression of angiogenic growth factors and their receptors [13,14,15,16]. Among MIA class antagonists, MIA-602 and MIA-690, which possess high receptor-binding affinity and weak inhibition of GH secretion [17,18], have demonstrated strong antitumor activity in multiple tumor types [14,17,19,20,21]. Furthermore, we have recently demonstrated that MIA-602 and MIA-690 can inhibit the growth of pleural mesothelioma both in vitro and in vivo while also enhancing the antitumor effects of chemotherapy drugs [22,23]. Additionally, MIA-602 showed strong anti-inflammatory and antioxidant properties in human THP-1-derived macrophages and peripheral blood mononuclear cells (PBMCs) stimulated with a combination of SARS-CoV-2 spike protein and lipopolysaccharide (LPS), suggesting a protective role against COVID-19-associated lung diseases [24].

Regarding the combination with radiotherapy, GHRH antagonist JMR-132 has previously been shown to confer radioprotection in mice when administered before low-dose whole-body radiation, while exhibiting a radiosensitizing effect at higher doses [25]. However, despite its ability to enhance chemotherapy [22,26], the combined anticancer effects of GHRH antagonists with radiotherapy remain unexplored. Thus, this study aimed to determine whether GHRH antagonists of MIA class enhance the antitumor effects of IR in vitro, in NSCLC cell lines and primary NSCLC cells, as well as to elucidate the underlying molecular mechanisms.

## 2. Results

### 2.1. MIA-602 and MIA-690 Reduce Viability and Proliferation in NSCLC Cells

**Figure 1:**
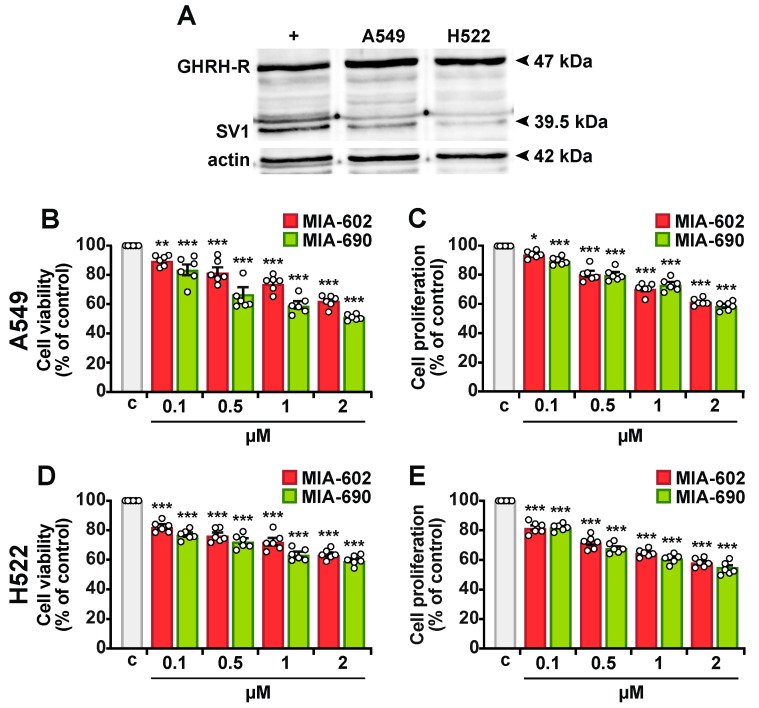
Expression of GHRH-Rs and inhibitory effect of MIA-602 and MIA-690 in NSCLC cells. (**A**) Western blot analysis of GHRH-R and SV1 in A549 and H522 cells. PC3 human prostate cancer cells served as a positive control (+), while actin was used as internal control. Cell viability and proliferation assessed by MTT and BrdU assays, respectively, in A549 (**B**,**C**) and H522 (**D**,**E**) cells, either untreated (c, control) or treated with MIA-602 or MIA-690 for 24 h at the indicated concentrations. Results, expressed as a percentage of control, are means ± SEM. * *p* < 0.05, ** *p* < 0.01, *** *p* < 0.001 vs. c, by one-way ANOVA and Dunnett’s multiple comparison post hoc test (*n* = 6).

We first verified the antitumor role of GHRH antagonists as single agents in A549 and H522 NSCLC cells. Western blot analysis revealed that both cell lines express GHRH-R and its splice variant SV1 (Figure 1A). The cells were then treated with increasing concentrations of MIA-602 and MIA-690 (0.1–2 μM) for 24 h. MTT and BrdU assay results showed that both peptides significantly and dose-dependently reduced cell viability and proliferation compared to the control in A549 (Figure 1B,C) and H522 cells (Figure 1D,E). These effects were statistically significant at all tested concentrations, with the greatest reduction observed at 2 μM. At this concentration, cell viability decreased to 37.43% and 36.25% with MIA-602 and to 48.44% and 40.29% with MIA-690 in A549 and H522 cells, respectively. Similarly, proliferation decreased to 38.58% and 41.22% with MIA-602 and to 41.38% and 44.27% with MIA-690 in A549 and H522 cells, respectively. Collectively, these findings suggest that MIA-602 and MIA-690 exhibit cytotoxic and antiproliferative effects in NSCLC cells.

### 2.2. MIA-602 and MIA-690 Increase the Sensitivity of NSCLC Cells to Ionizing Radiation

**Figure 2:**
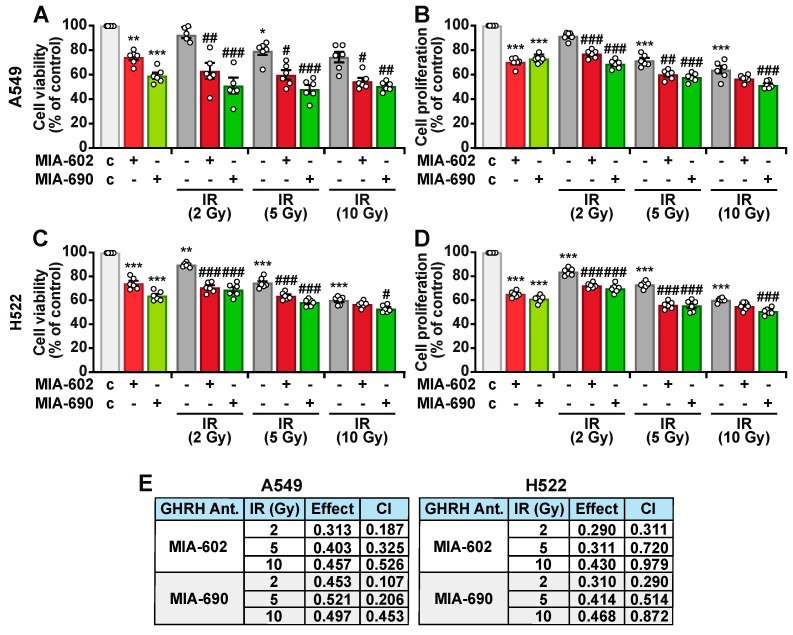
Radiosensitizing effect of MIA-602 and MIA-690 in NSCLC cells. Cell viability and proliferation assessed by MTT and BrdU assays, respectively, in A549 (**A**,**B**) and H522 (**C**,**D**) cells, either left untreated (c, control) or treated with the indicated doses of IR, alone or in combination with 1 μM MIA-602 or MIA-690 for 24 h. Results, expressed as percentage of control, are means ± SEM. * *p* < 0.05, ** *p* < 0.01, *** *p* < 0.001 vs. c; ^#^ *p* < 0.05, ^##^ *p* < 0.01, ^###^ *p* < 0.001 vs. the corresponding IR dose by one-way ANOVA and Tukey’s multiple comparison post hoc test (*n* = 6). (**E**) Combination index (CI) values, calculated with CompuSyn software (www.combosyn.com), for 1 μM MIA-602 or MIA-690 with the indicated IR doses on cell viability inhibition in A549 and H522 cells.

To further assess the ability of GHRH antagonists to sensitize NSCLC cells to IR, A549 and H522 cells were either left untreated or exposed to single doses of IR (2, 5, or 10 Gy). Following irradiation, the cells were incubated for 24 h with 1 μM MIA-602 or MIA-690, a concentration determined based on prior dose–response experiments. MIA-602 and MIA-690 individually reduced cell viability and proliferation to a similar extent in both cell lines (Figure 2A–D). Additionally, IR treatment alone led to a reduction in cell viability and proliferation compared to the control, with effects observed at doses of 5 and 10 Gy in A549 cells (Figure 2A,B), and 2, 5, and 10 Gy in H522 cells (Figure 2C,D). The combination of MIA-602 or MIA-690 with IR further decreased cell viability and proliferation beyond the effects of either treatment alone, with the most pronounced reduction seen with MIA-690 at 5 Gy in A549 cells. Chou–Talalay analysis confirmed a synergistic interaction between MIA-602 or MIA-690 and IR across all radiation doses in both cell lines, as indicated by combination index (C.I.) values of <1 (Figure 2E).

Overall, these results suggest that GHRH antagonists enhance the cytotoxic effects of IR in lung cancer cells.

### 2.3. MIA-690 Reduces the Expression of GHRH Receptors and IGF1 in A549 Cells Exposed to IR

**Figure 3:**
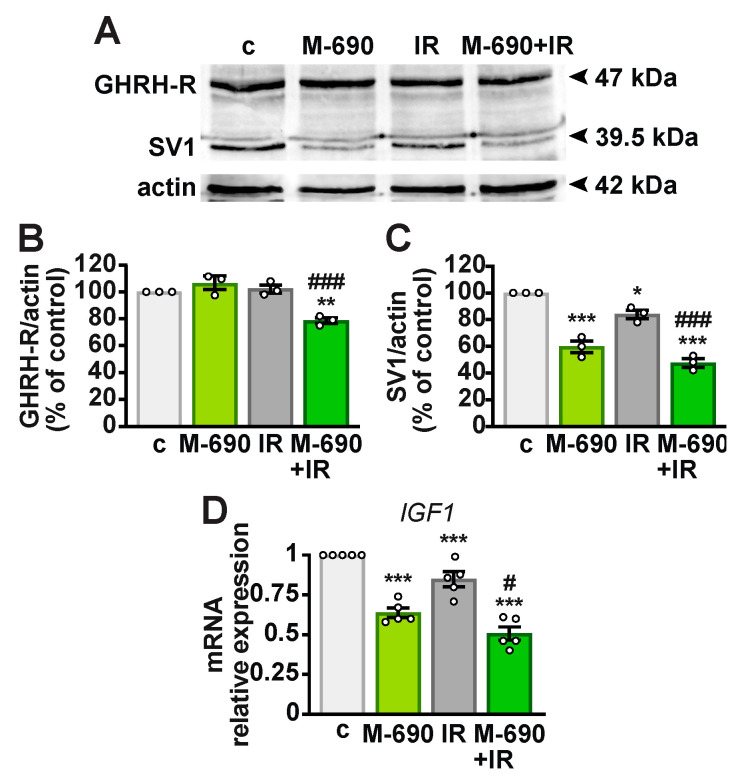
Reduction in GHRH-Rs and *IGF1* expression in A549 cells treated with MIA-690 and IR combination. (**A**) Representative immunoblot analysis of GHRH-R and SV1 after 24 h of treatment. Cells were either untreated (c, control) or exposed to 5 Gy IR, 1 μM MIA-690 (M-690), or their combination. Graphs show the densitometric quantification of GHRH-R (**B**) and SV1 (**C**) normalized to actin and expressed as a percentage of the control. Data are means ± SEM. * *p* < 0.05, ** *p* < 0.01, *** *p* < 0.001 vs. c; ^###^ *p* < 0.001 vs. IR, by one-way ANOVA and Tukey’s multiple comparison post hoc test (*n* = 3). (**D**) IGF1 mRNA levels were measured by real-time PCR, normalized to *18S rRNA*, and expressed as means ± SEM. *** *p* < 0.001 vs. c; ^#^ *p* < 0.05 vs. IR, by one-way ANOVA and Tukey’s multiple comparison post hoc test (*n* = 5).

Given its slightly greater effect compared to MIA-602, MIA-690 was selected for further investigation. GHRH-R, particularly its splice variant SV1, plays a role in the antitumor activities of GHRH antagonists [18,20]. Therefore, we examined whether MIA-690 affects GHRH-R expression in NSCLC cells. Western blot analysis showed that GHRH-R protein levels remained unchanged in A549 cells treated with either MIA-690 or IR alone, but were reduced when both were combined, compared to control (21.34%) and IR alone (23.34%) (Figure 3A,B). SV1 expression was downregulated by MIA-690 (41%) and by IR (16%), with a more pronounced decrease observed in the combined treatment (52.34% vs. control and 36.34% vs. IR). We also measured IGF1 mRNA levels, as its reduction is linked to the anticancer effects of GHRH antagonists [18,22]. Both MIA-690 and IR independently decreased *IGF1* expression, with an enhanced effect observed in combination (Figure 3D). These findings indicate that the radiosensitizing properties of MIA-690 are associated with the downregulation of GHRH-Rs and *IGF1*.

### 2.4. MIA-690 Inhibits Cell Growth and Regulates the Cell Cycle in Irradiated NSCLC Cells

**Figure 4:**
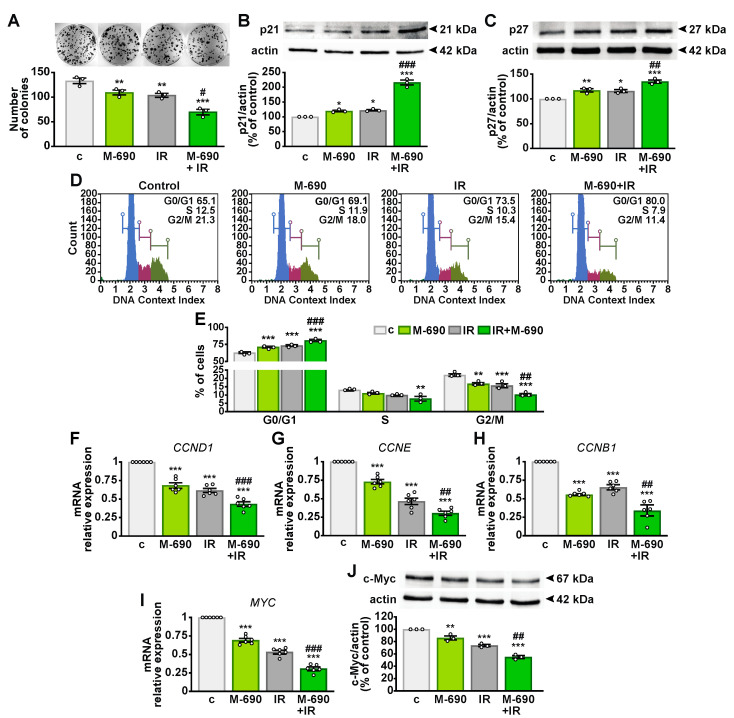
Inhibitory effects of MIA-690 and IR combination on colony formation and cell cycle regulators. (**A**) Representative colony formation assay in A549 cells, either untreated (c, control) or cultured for 10 days following exposure to a single dose of 5 Gy IR and 1 μM MIA-690 (M-690), (*n* = 3). Representative immunoblot analysis of p21 (**B**) and p27 (**C**) protein expression (top panels) in A549 cells treated for 24 h with 1 µM MIA-690, 5 Gy IR, or their combination. Actin was used as a loading control (bottom panels). Graphs represent densitometric analysis normalized to actin and expressed as a percentage of control. Data (**A**–**C**) are means ± SEM (*n* = 3). * *p* < 0.05, ** *p* < 0.01, *** *p* < 0.001 vs. c; ^#^ *p* < 0.05, ^##^ *p* < 0.01, ^###^ *p* < 0.001 vs. IR, by one-way ANOVA and Tukey’s post hoc test. (**D**) Representative flow cytometry images illustrating cell cycle distribution [G0/G1 (blue), S (magenta), and G2/M (green) phases] in A549 cells treated for 24 h with 1 μM MIA-690, 5 Gy IR, or their combination, using the Muse™ Cell Cycle kit. (**E**) Quantification of the percentage of cells in each phase, expressed as mean ± SEM (*n* = 3). ** *p* < 0.01, *** *p* < 0.001 vs. c; ^##^ *p* < 0.01, ^###^ *p* < 0.001 vs. IR in each phase, by two-way ANOVA and Tukey’s post hoc test. (**F**–**I**) mRNA expression levels of cyclin D1 (*CCND1*) (**F**), cyclin E (*CCNE*) (**G**), cyclin B1 (*CCNB1*) (**H**), and *MYC* (**I**) assessed by real-time PCR in A549 cells treated for 24 h with 1 μM MIA-690, 5 Gy IR, or their combination. Expression levels are normalized to *18S rRNA* and are means ± SEM (*n* = 6). *** *p* < 0.001 vs. c; ^##^ *p* < 0.01, ^###^ *p* < 0.001 vs. IR, by one-way ANOVA and Tukey’s post hoc test. (**J**) Representative immunoblot analysis of c-Myc expression (top panel) in A549 cells treated for 24 h with 1 μM MIA-690, 5 Gy IR, or their combination. Actin was used as a loading control (bottom panel). The graph shows densitometric analysis normalized to actin and expressed as a percentage of control. Data are means ± SEM (*n* = 3). ** *p* < 0.01, *** *p* < 0.001 vs. c; ^##^ *p* < 0.01 vs. IR, by one-way ANOVA and Tukey’s post hoc test.

To further confirm the radiosensitizing effect of MIA-690 on cell viability, we evaluated the clonogenic potential of A549 using the clonogenic assay, the gold standard for testing radiation modifiers [25]. Both MIA-690 and IR individually reduced colony formation, with their combination exerting an even stronger inhibitory effect (Figure 4A). Additionally, MIA-690 and IR significantly increased the protein levels of the cyclin-dependent kinase inhibitors (CDKIs) p21 and p27 (Figure 4B,C), which were already elevated by the single treatments.

To explore whether the antiproliferative effects of MIA-690 were linked to cell cycle arrest, we performed flow cytometry analysis. The combined treatment with MIA-690 and IR resulted in a higher proportion of cells in the quiescent G0/G1 phase while decreasing the G2/M population compared to single treatments (Figure 4D,E). Consistently, transcript levels of *CCND1* and *CCNE* (encoding cyclin D1 and E) and *CCNB1* (encoding cyclin B1) were significantly reduced following MIA-690 and IR treatment compared with IR alone (Figure 4F–H). Moreover, the expression of the *MYC* oncogene and its corresponding protein c-Myc, both attenuated by individual treatments, was further suppressed by their combination (Figure 4I,J). These findings suggest that MIA-690 enhances the antitumor effects of IR by inhibiting cell cycle progression and modulating key cell cycle regulators.

### 2.5. MIA-690 Sensitizes IR-Induced Apoptosis in NSCLC Cells

**Figure 5:**
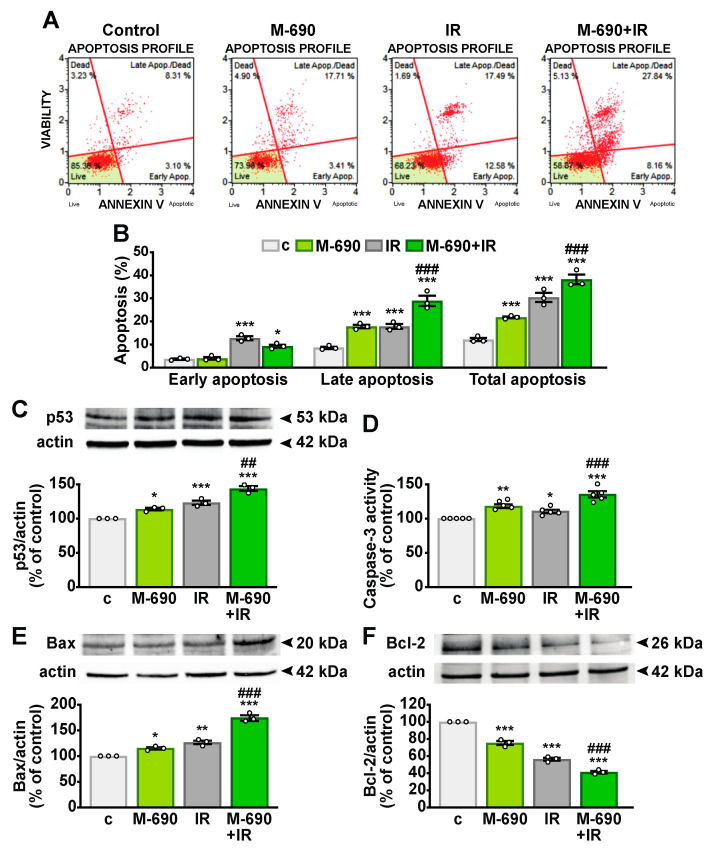
Proapoptotic effect of MIA-690 and IR combination in NSCLC cells. A549 cells were either left untreated (c, control) or treated with 1 μM MIA-690 (M-690) and/or exposed to 5 Gy IR, alone or in combination, for 24 h. (**A**) Representative flow cytometry results using the Muse™ Annexin V and Dead Cell assay in A549 cells. (**B**) Quantification of early apoptosis, late apoptosis, and total apoptosis (early + late) in Annexin V-stained cells. Results are means ± SEM. * *p* < 0.05 and *** *p* < 0.001 vs. c; ^###^ *p* < 0.001 vs. IR, by two-way ANOVA and Tukey’s post hoc test. (**C**) Representative Western blot of p53 protein (top), with actin used as loading control (bottom). (**D**) Apoptosis assessment via caspase-3 activity. (**E**,**F**) Western blot analysis of Bax (**E**) and Bcl-2 (**F**) expression. Graphs show the densitometric analysis of p53 (**C**), Bax (**E**), and Bcl-2 (**F**) levels, normalized to actin, and expressed as a percentage of the control. Data are means ± SEM. * *p* < 0.05, ** *p* < 0.01, and *** *p* < 0.001 vs. c; ^##^ *p* < 0.01, ^###^ *p* < 0.001 vs. IR, by one-way ANOVA and Tukey’s post hoc test (*n* = 3 for **B**,**C**,**E**,**F**; *n* = 5 for **D**).

Radiation therapy induces apoptosis as one of its primary biological effects [26]. To determine whether MIA-690 amplifies this proapoptotic response in A549 cells, we analyzed apoptosis levels using flow cytometry with Annexin V staining. The combination of MIA-690 and IR significantly increased the proportion of cells undergoing late apoptosis compared to IR alone (Figure 5A,B). Additionally, the tumor suppressor protein p53 was upregulated following treatment with either MIA-690 or IR individually, with a markedly greater increase observed in the combined treatment (Figure 5C). Consistently, MIA-690 enhanced the activation of caspase-3 (Figure 5D) and elevated the proapoptotic Bax levels (Figure 5E) in irradiated cells, while concurrently reducing antiapoptotic Bcl-2 expression (Figure 5F). These results suggest that MIA-690 strengthens the proapoptotic effects of radiotherapy in NSCLC cells.

### 2.6. MIA-690 Mitigates Radioresistance in NSCLC Cells

**Figure 6:**
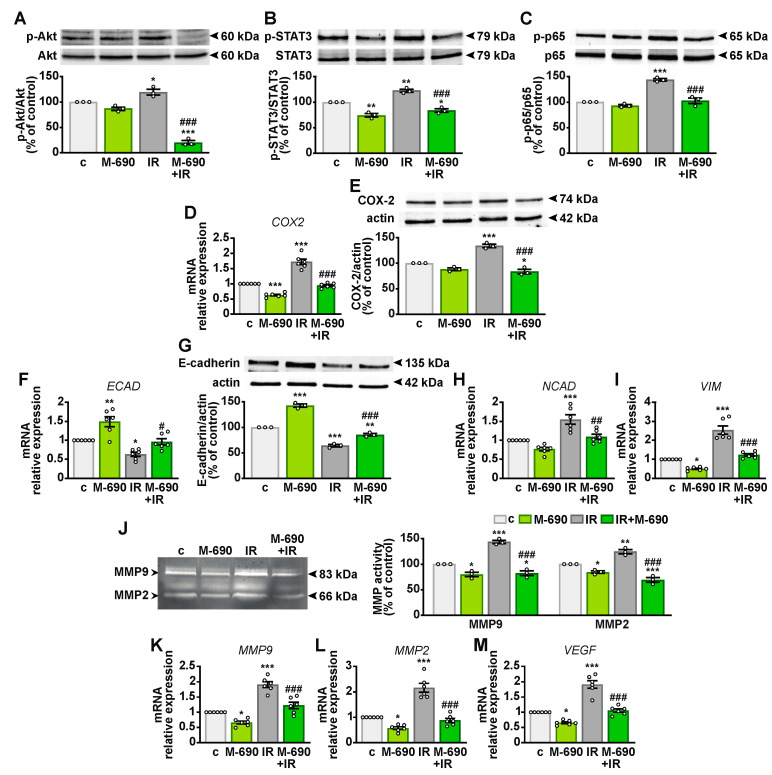
Inhibitory effects of MIA-690 on radioresistance and radiation-induced EMT in A549 cells. Cells were either untreated (c, control), treated with 1 μM MIA-690 (M-690), exposed to 5 Gy IR, or subjected to a combination of both treatments for 24 h. (**A**–**C**) Representative Western blots showing phosphorylated Akt (p-Akt) (**A**), STAT3 (p-STAT3) (**B**), and p65 (p-p65) (**C**) (top panels). Blots were reprobed with antibodies against total proteins for normalization (bottom panels), with results expressed as percentage of the control (*n* = 3). (**D**) *COX2* mRNA levels assessed by real-time PCR and normalized to *18S rRNA* (*n* = 6). (**E**) COX-2 protein expression analyzed by Western blot (top panel), with actin used as a loading control (bottom panel) (*n* = 3). (**F**) E-cadherin (*ECAD*) mRNA levels assessed by real-time PCR and normalized to *18S rRNA* (*n* = 6). (**G**) Western blot analysis of E-cadherin expression (top panel), with actin used as a loading control (bottom panel), (*n* = 3). Densitometric analysis of COX-2 (**E**) and E-cadherin (**G**) protein levels, normalized to actin and expressed as a percentage of the control, is shown in the accompanying graphs. (**H**,**I**) mRNA levels of N-cadherin (*NCAD*) (**H**) and vimentin (*VIM*) (**I**) measured by real-time PCR and normalized to *18S rRNA* (*n* = 6). (**J**) Representative gelatin zymography of MMP9 and MMP2 activity in A549-conditioned medium. The graph presents densitometric analysis of MMP9 and MMP2 activity as a percentage of the control (*n* = 3). (**K**–**M**) *MMP9* (**K**), *MMP2* (**L**), and *VEGF* (**M**) levels analyzed by real-time PCR and normalized to *18S rRNA* (*n* = 6). Data are means ± SEM. * *p* < 0.05, ** *p* < 0.01, and *** *p* < 0.001 vs. c; ^#^ *p* < 0.05, ^##^ *p* < 0.01, and ^###^ *p* < 0.001 vs. IR, by one-way ANOVA and Tukey’s post hoc test.

To further explore the radiosensitizing effects of MIA-690 in A549 cells, we investigated the mechanisms underlying radioresistance, including cell viability, inflammatory pathways, angiogenesis, invasion, and EMT [5]. Although MIA-690 alone had no significant effect, it effectively suppressed IR-induced phosphorylation of Akt (Figure 6A). Similarly, MIA-690 reduced the activation of signal transducer and activator of transcription 3 (STAT3) both independently and in irradiated cells (Figure 6B). Additionally, while MIA-690 alone had no impact, it prevented IR-induced phosphorylation of the NF-κB subunit p65 (Figure 6C).

MIA-690 also counteracted IR-induced expression of cyclooxygenase-2 (COX-2) at both mRNA and protein levels, demonstrating an inhibitory effect even in the absence of irradiation (Figure 6D,E). EMT, characterized by the downregulation of epithelial markers like E-cadherin and the upregulation of mesenchymal markers such as N-cadherin, vimentin, and matrix metalloproteinases (MMPs) [27], was disrupted by MIA-690. IR exposure reduced *ECAD* transcript levels and its corresponding protein, E-cadherin, while upregulating *NCAD* and *VIM*. MIA-690 reversed these effects by increasing E-cadherin expression and suppressing mesenchymal gene expression, even in the absence of irradiation (Figure 6F–I). Furthermore, gelatin zymography analysis of A549 conditioned medium revealed that MIA-690, both alone and in irradiated cells, inhibited IR-induced MMP9 and MMP2 activity (Figure 6J). Consistently, IR elevated the *MMP9* and *MMP2* mRNA levels, whereas MIA-690 exerted an inhibitory effect both independently and in combination with IR (Figure 6K,L). Furthermore, the radiation-induced increase in *VEGF* was attenuated by MIA-690, which also showed an inhibitory effect alone (Figure 6M). Collectively, these findings indicate that MIA-690 disrupts multiple signaling pathways involved in radioresistance.

### 2.7. MIA-690 and MIA-602 Enhance the Sensitivity of Primary NSCLC Cells to IR

**Figure 7:**
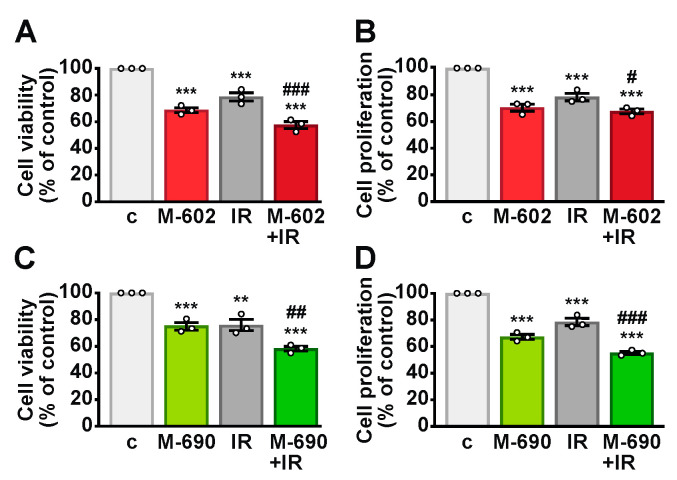
Radiosensitizing effect of GHRH antagonists in primary NSCLC cells. Cell viability (**A**,**C**) and proliferation (**B**,**D**) assessed by MTT and BrdU assay, respectively, in untreated control cells (c) and in cells exposed to 5 Gy IR followed by treatment for 24 h with 1 μM MIA-602 (M-602) (**A**,**B**) or MIA-690 (M-690) (**C**,**D**), either alone or in combination. Results, expressed as a percentage of the control, are means ± SEM. ** *p* < 0.01, *** *p* < 0.001 vs. c; ^#^ *p* < 0.05, ^##^ *p* < 0.01, ^###^ *p* < 0.001 vs. IR, by one-way ANOVA and Tukey’s multiple comparison post hoc test (*n* = 3).

The potential radiosensitizing effects of GHRH antagonists were evaluated in primary NSCLC cells derived from human lung adenocarcinoma tumors. Cells were treated individually with MIA-690, MIA-602, or IR (5 Gy), as well as in combination. In line with findings from NSCLC cell lines, both antagonists reduced cell viability (Figure 7A,C) and proliferation (Figure 7B,D). Moreover, the combined treatment demonstrated greater efficacy than IR alone. These results indicate that MIA-602 and MIA-690 not only exert inhibitory effects independently but also enhance the antitumor activity of IR in primary lung cancer cells.

## 3. Discussion

Radiotherapy is a primary treatment for advanced lung cancer; however, radioresistance often occurs, leading to therapy failure and local recurrence. Additionally, high radiation doses can cause adverse effects, contributing to a poor prognosis [5,28,29]. Therefore, developing radiosensitizers is crucial to reduce the radiation exposure, minimize damage to healthy tissues, and mitigate drug toxicity. In this study, we demonstrate that GHRH antagonists MIA-602 and MIA-690 not only exert individual inhibitory effects but also enhance the sensitivity of NSCLC cell lines and primary NSCLC cells to radiation therapy. This occurs through the suppression of cell growth, promotion of apoptosis, and inhibition of signaling pathways linked to radioresistance.

GHRH and its receptors (GHRH-Rs) have been identified in both normal lung and lung cancer tissues [15,30,31]. Consistently, we detected the expression of GHRH-R and its splice variant SV1 in A549 cells and, for the first time, in H522 NSCLC cells. Notably, high GHRH-R expression in rectal tumors has been associated with poor tumor regression following neoadjuvant chemoradiotherapy, highlighting the potential of targeting GHRH-R with antagonists in patients who respond poorly to treatment [32]. Furthermore, GHRH antagonists have been shown to enhance chemotherapy efficacy in various cancers, including lung cancer, pleural mesothelioma, and pituitary adenomas [13,15,20,22,23,33,34]. While the antitumor effects of MIA-602 and MIA-690 in lung cancer have been previously described, this is the first study to provide evidence of their radiosensitizing properties. To our knowledge, only one prior study reported that pretreatment with an earlier GHRH antagonist, JMR-132, conferred radioprotection at low radiation doses (<10 Gy) while enhancing radiosensitivity at higher doses (>10 Gy) [35]. These findings, along with the well-established antitumor activity of GHRH antagonists, motivated our investigation into the radiosensitizing effects of MIA-602 and MIA-690 in NSCLC.

Our results indicate that both antagonists independently reduced cell viability and proliferation in NSCLC cell lines and primary NSCLC cells. Most importantly, their inhibitory effects on cell viability were significantly amplified when combined with radiation, demonstrating a synergistic enhancement of radiation response. Notably, the combined effects of GHRH antagonists and IR exceeded those observed with higher radiation doses alone in both cell lines. This suggests that combining GHRH antagonists with radiation could enhance therapeutic efficacy at lower radiation doses, potentially minimizing the side effects associated with radiotherapy. Our findings also indicate that compared to H522 cells, A549 cells exhibit greater sensitivity to GHRH antagonists, both alone and in combination with IR. This may be partly attributed to the slightly higher expression of SV1 in A549 cells. Furthermore, KRAS mutations in NSCLC have been linked to aberrant activation of the PI3K/Akt and NF-κB pathways [36,37], as well as increased expression of COX-2 [37]. Notably, previous studies have demonstrated that GHRH antagonists can downregulate KRAS and COX-2 expression while inhibiting PI3K/Akt and NF-κB signaling in NSCLC models [13,31]. Consistent with these observations, this study shows that MIA-690 counteracts IR-induced COX-2 expression and the phosphorylation of Akt and NF-κB, suggesting that A549 cells may exhibit enhanced responsiveness to GHRH antagonists in a KRAS-dependent manner. However, further investigations using different cell lines and human primary cells with distinct oncogenic mutations are warranted.

GHRH antagonists exert their antitumor activity by binding to GHRH-R and SV1, thereby disrupting the autocrine/paracrine mitogenic signaling mediated by tumor-derived GHRH, IGF1, and IGF2 [18,20,38]. Additionally, these antagonists reduce the production of tumoral IGF1 [22,23,39], a key player in tumorigenesis and cancer progression [40]. However, MIA-class GHRH antagonists, particularly MIA-602 and MIA-690, exhibit high GHRHR binding affinity while exerting minimal GH inhibitory activity [17,20]. Previous studies by our group and others have shown that treatment with MIA-602 and MIA-690 suppresses tumoral IGF1 expression in mouse models of human cancers without significantly altering serum GH levels, further supporting the GH-independent actions of these peptides [18,22,41]. Interestingly, MIA-602 has also been reported to exert anti-inflammatory effects by inhibiting GH and IGF-1 production in rat ocular epithelial cells [42]. Therefore, it remains possible that MIA-class antagonists act locally in lung tumors to inhibit the autocrine and paracrine effects of locally produced GH. In contrast, the previous class of antagonists, MZ-4-71 and JV-1-36, were found ineffective in reducing GH expression in human GH-secreting pituitary adenoma cells [41]. Further research is required to explore the potential effects of GHRH antagonists on local GH production in lung tumors and other cancer types. Importantly, SV1 has been shown to promote mitogenic effects in tumor cells independent of ligand binding, making it a promising therapeutic target in cancer [18,43,44]. In this study, MIA-690 not only decreased SV1 expression in A549 cells but also enhanced the IR-induced suppression of SV1. Furthermore, the combination of MIA-690 and IR proved more effective than either treatment alone in reducing GHRH-R and IGF1 levels. The cell death mechanisms triggered by irradiation involve cell cycle arrest and apoptosis, facilitated by the generation of hydroxyl radicals, double-strand breaks, and activation of ataxia-telangiectasia mutated (ATM), which in turn promotes p53 phosphorylation and the expression of cyclin-dependent kinase inhibitors (CDKIs) p21 and p27. These CDKIs regulate cell cycle checkpoints, leading to arrest in the G1/S and G2/M phases [45]. GHRH antagonists have been shown to inhibit cell cycle progression in several cancers by upregulating p21 and p27 while downregulating cyclins [13,15,22,23,46,47]. Notably, p21 and p27 are frequently dysregulated in various tumors, and their loss has been linked to an increased risk of NSCLC recurrence and mortality [48]. In addition to suppressing cell growth, we found that MIA-690 upregulated p21 and p27, both as a monotherapy and, more significantly, in combination with IR. Consistent with this, MIA-690 enhanced the IR-induced inhibition of cell cycle progression and further downregulated cyclins D1 and E, which drive the G1/S transition, as well as cyclin B1, which governs the G2/M phase [48].

The *MYC* oncogene is activated in most cancers, where it enhances cancer cell-intrinsic processes such as proliferation, metabolism, invasion, apoptosis, and autophagy, while inhibiting protective mechanisms like differentiation and senescence. Inactivation of *MYC* can lead to sustained tumor regression and increased radiosensitivity, whereas its overexpression promotes radioresistance by driving EMT, tumor angiogenesis, and invasion [49]. In this study, MIA-690 and IR individually reduced *MYC* expression at both mRNA and protein levels, with their combination leading to an even greater decrease. This aligns with previous findings showing that GHRH antagonists lower *MYC* expression in various experimental cancer models, including when combined with chemotherapy [19,22,23,34].

p53 functions as a tumor suppressor by inducing cell cycle arrest, senescence, apoptosis, and DNA repair. Beyond these canonical roles, p53 also promotes antioxidant responses, ferroptosis, and the inhibition of angiogenesis, invasion, and metastasis, while modulating immune responses, making it a promising target for novel anticancer therapies [50]. In this study, MIA-690 not only increased p53 levels on its own but also enhanced the IR-induced upregulation of p53. Furthermore, MIA-690 sensitized cancer cells to IR-induced apoptosis by increasing annexin staining, caspase-3 activity, and Bax protein expression while downregulating the antiapoptotic protein Bcl-2. Notably, while the proapoptotic effects of GHRH antagonists in tumors are well established, their ability to enhance apoptosis in combination with radiotherapy has not been previously reported.

Epidermal growth factor receptor (EGFR) signaling plays a key role in tumor progression and radioresistance. Additionally, IR can trigger ligand-independent activation of EGFR and its downstream pathways, STAT3 and PI3K/Akt [5]. Akt functions as a proliferative and antiapoptotic kinase by phosphorylating p21 and p27, while also activating NF-κB, which drives the transcription of inflammatory genes [5,45]. Consequently, targeting the PI3K/Akt pathway may enhance tumor sensitivity to IR and mitigate radioresistance. Our findings reveal that MIA-690 effectively reduces Akt phosphorylation in response to IR. Similarly, MIA-690 counteracted IR-induced phosphorylation of STAT3 and NF-κB, which, in lung cancer, has been linked to the upregulation of COX-2, MMP9, and cyclin D1, factors that contribute to increased cancer cell survival, proliferation, invasion, and EMT [51,52]. Moreover, MIA-690 not only suppressed COX-2 expression at both mRNA and protein levels but also inhibited IR-induced COX-2 upregulation. Consistently, MIA-602 and MIA-690 have been reported to suppress STAT3 activity in SCLC and NSCLC cell lines in vitro [15] and in pleural mesothelioma in vivo [22] by inhibiting NF-κB, COX-2, and MMPs, both alone and in combination with chemotherapy drugs. Additionally, recent findings indicate that MIA-602 mitigates inflammation induced by the SARS-CoV-2 spike protein and LPS in human THP-1 macrophages and PBMCs by inhibiting NF-κB, STAT3, COX-2, and MMP9 [24]. Proinflammatory factors such as NF-κB can upregulate COX-2 activity, leading to tumor immune suppression, increased inflammation, apoptosis resistance, and EMT [53]. Moreover, COX-2 has been implicated in enhancing radioresistance in A549 cells through its metabolite prostaglandin E2 [54]. Persistent STAT3 activation, driven by cytokines, growth factors, and mutations in EGFR and SRC kinases, has been observed in over 50% of NSCLC patients and is associated with chemoresistance, radioresistance, and immune evasion [55].

EMT is a key process contributing to radioresistance, characterized by the downregulation of epithelial adhesion markers such as E-cadherin and the upregulation of mesenchymal markers like N-cadherin and vimentin [27]. EMT is governed by multiple signaling pathways, transcription factors, enzymes, and growth factors that drive cell proliferation, inflammation, angiogenesis, and invasion, ultimately facilitating cancer progression [27]. Notably, the loss of E-cadherin has been linked to tumor progression, metastasis, and recurrence in a mouse model of lung adenocarcinoma [56]. Consequently, strategies to counteract radioresistance could enhance cancer prognosis. Our findings demonstrated that MIA-690, both alone and in combination with ionizing radiation (IR), inhibited EMT by upregulating *ECAD* expression and its corresponding protein, E-cadherin, while downregulating *NCAD* and *VIM*. Additionally, MIA-690 suppressed the IR-induced increase in MMP9 and MMP2 transcript levels and activity, as well as reducing *VEGF* expression. The tumor microenvironment, particularly endothelial cells and tumor vasculature, plays a crucial role in radioresistance. IR-induced vascular damage exacerbates tumor hypoxia, thereby stimulating angiogenic factors such as VEGF and MMPs [29]. In line with this, GHRH antagonists of the MIA class have been shown to impede cancer cell migration and invasion by preserving E-cadherin expression and inhibiting MMP2 and MMP9 expression and activity [15,57]. Similarly, MIA-602 and MIA-690 exhibited antitumor effects in pleural mesothelioma by reducing MMP and VEGF levels both in vitro and in vivo [22,23].

In summary, our study demonstrates that MIA-class GHRH antagonists enhance the sensitivity of human NSCLC cell lines and primary NSCLC cells to the antitumor effects of IR while suppressing molecular pathways associated with radioresistance. While additional studies are needed to validate these findings across various experimental models, our results indicate that GHRH antagonists hold potential for development as adjuncts to radiotherapy in NSCLC and potentially other cancers.

## 4. Materials and Methods

### 4.1. Reagents

GHRH-R antagonists MIA-602 [(PhAc-Ada)^0^-Tyr^1^, d-Arg^2^, Fpa5^6^, Ala^8^, Har^9^, Tyr(Me)^10^, His^11^, Orn^12^, Abu^15^, His^20^, Orn^21^, Nle^27^, d-Arg^28^, Har^29^]hGH-RH(1–29)NH_2_, and MIA-690 [(PhAc-Ada)^0^-Tyr^1^, d-Arg^2^, Cpa^6^, Ala^8^, Har^9^, Fpa5^10^, His^11^, Orn^12^, Abu^15^, His^20^, Orn^21^, Nle^27^, d-Arg^28^, Har^29^]hGH-RH(1–29)NH_2_ were synthesized and purified by Dr. Renzhi Cai and Dr. Andrew V. Schally at the Veterans Affairs Medical Center, University of Miami, Miami, FL [17]. MIA-602 and MIA-690 were dissolved in 100% dimethyl sulfoxide (DMSO) (Sigma-Aldrich; Merck, Milan, Italy) and diluted with the appropriate incubation medium. DMSO concentration never exceeded 0.1% (vol/vol). Dulbecco’s Modified Eagle’s Medium (DMEM), fetal bovine serum (FBS), bovine serum albumin (BSA), penicillin, amphotericin B, streptomycin, L-glutamine, collagenase IV, hydrocortisone, insulin, estradiol, progesterone and epidermal growth factor (EGF), 3-(4,5-dimethylthiazol-2-yl)-2,5-diphenyl-2H-tetrazolium bromide (MTT), primers and cell culture reagents were obtained from Sigma-Aldrich (Merck, Milan, Italy). RT-PCR and real-time PCR reagents were obtained from Invitrogen (ThermoFisher Scientific, Milan, Italy). The antibody for GHRH-R (ab28692, RRID:AB_732729), which also recognizes SV1, was obtained from Abcam (Waltham, MA, USA). p-Akt (9271S, RRID:AB_329825), Akt (4685S, RRID:AB_2225340), p-Stat3 (Tyr705) (9131S, RRID:AB_331586), Stat3 (30835S, RRID:AB_2798995), p-NF-κB p65 (Ser536) (3033S, RRID:AB_331284), NF-κB p65 (8242S, RRID:AB_10859369), p27 (2552S, RRID:AB_10693314), COX-2 (12282, RRID:AB_2571729), E-cadherin (14472, RRID:AB_2728770) and Bax (5023S, RRID:AB_10557411) antibodies were obtained from Cell Signaling Technology (Danvers, MA, USA). p53 (sc-1313, RRID:AB_632148), p21 (sc-56335, RRID:AB_785023), Bcl-2 (sc-783, RRID:AB_2243455), c-Myc (sc-40, RRID:AB_627268), and actin (sc-376421, RRID:AB_11149557) antibodies were obtained from Santa Cruz Biotechnology (Dallas, TX, USA).

### 4.2. Cell Culture and Treatments

A549 (RRID:CVCL_0023) and NCI-H522 (RRID:CVCL_1567) NSCLC cell lines were purchased from the Italian Biobank of Veterinary Resources (IBVR), Brescia, Italy. The cells were maintained at 37 °C in a 5% CO_2_ humidified atmosphere in DMEM with 10% FBS, L-glutamine (2 mM), penicillin (100 U/mL), streptomycin (100 μg/mL), and amphotericin B (250 ng/mL). Irradiation was performed with a Linear accelerator Elekta^©^ Sinergy (Elekta Solution, Stockholm, Sweden) at the Radiotherapy Unit, A.O.U. Città della Salute e della Scienza (Torino, Italy), using a 6 MV photon beam in homogeneous conditions; the dose rate was set at 3 Gy/min. MIA-602 or MIA-690 (1 μM) were added to serum-deprived medium immediately after irradiation, and cells were incubated for 24 h at 37 °C in a 5% CO_2_ humidified atmosphere.

### 4.3. Isolation of Primary Lung Cancer Cells

Human lung cancer tissues were obtained from 3 male patients with histologically confirmed resectable stage I-IIIA NSCLC and enrolled in a prospective observational study (PROMOLE) approved by the Ethical Committee of A.O.U. San Luigi Gonzaga (Orbassano, Italy) (protocol n.73/2018, amendment n. 2866/2023) and conducted in accordance with the Declaration of Helsinki. Written informed consent was obtained from all participants before surgery. Tissues were immediately placed into ice-cold RPMI-1640 supplemented with penicillin (100 U/mL), streptomycin (100 μg/mL), and amphotericin B (250 ng/mL). After blood clot removal, samples were rinsed with sterile phosphate-buffered saline (PBS), cut into small fragments then incubated with collagenase IV (final concentration 0.5%) (Sigma-Aldrich; Merck, Milan, Italy) for 1 h at 37 °C. Cell debris was removed through a 40 μm diameter cell mesh filter (Sigma-Aldrich; Merck, Milan, Italy) and centrifuged for 15 min at 400× *g*. NSCLC cells were cultured at 37 °C and 5% CO_2_ in DMEM/F-12 supplemented with 10% FBS, penicillin (100 U/mL), streptomycin (10 μg/mL), amphotericin B (10 mg/mL), L-glutamine (2 mM), hydrocortisone (1 μg/mL), insulin (10 mg/mL), estradiol (20 μg/mL), progesterone (1 mg/mL), and EGF (50 μg/mL).

### 4.4. Cell Viability and Proliferation

Cells were seeded in 96-well plates at a concentration of 3 × 10^3^ cells/well. After 48 h, cells were serum-starved for 12 h and exposed to the different stimuli for a further 24 h. Cell viability was assessed by MTT assay (Sigma-Aldrich; Merck, Milan, Italy, #M5655), as previously described [22]. Cell proliferation was evaluated using the 5-bromo-2-deoxyuridine (BrdU) incorporation ELISA kit (Roche Diagnostic, Milan, Italy, #11647229001). Cell viability and proliferation were examined by colorimetric analysis at 570 nm and 450 nm absorbance, respectively, using the LT-4000 microplate reader (Euroclone, Milan, Italy).

### 4.5. Caspase-3 Activity

Cells were seeded into a 6-well plate at a concentration of 3 × 10^4^ cells/well. Caspase-3 activity was assessed in cell lysates by a Caspase-3 Colorimetric Assay Kit (Abcam, Waltham, MA, USA, #ab39401) according to the manufacturer’s instructions. Briefly, cells were resuspended in cell lysis buffer, incubated for 10 min at 4 °C, centrifuged, and cytosolic extracts were used for protein quantification. Samples were then incubated for 2 h with DEVD-pNA substrate. Colorimetric analysis was performed at 405 nm absorbance using the LT-4000 microplate reader (Euroclone, Milan, Italy).

### 4.6. Colony Formation

A549 cells were seeded into 6-well plates at a concentration of 3 × 10^2^ cells/well, untreated or exposed to IR, then cultured for 10 days in DMEM with 10% FBS in either the presence or absence of MIA-690 (1 μM). Cells were subsequently fixed with methanol, colonies stained with crystal violet (0.05%), and plates photographed using a digital camera (ChemiDoc XRS, BioRad, Milan, Italy). Colonies were counted using Image J software (https://imagej.nih.gov, RRID:SCR_003070).

### 4.7. Cell Cycle Analysis

Cell cycle analysis was performed using the Muse^TM^ Cell Cycle Kit (Luminex, Austin, TX, USA) according to the manufacturer’s instructions. Briefly, 1 × 10^5^ cells were seeded in 60 mm dishes and after 48 h exposed to the different stimuli in medium supplemented with 2% FBS for a further 24 h. Cells were then detached with PBS 1X/EDTA (5 mM), centrifuged (300× *g*, 5 min), and fixed with pre-cooled ethanol. The cells were then treated with Muse^TM^ Cell Cycle Reagent for 30 min and analyzed with Muse^TM^ Cell Analyzer Software _V1.5.0.0 (Luminex, Austin, TX, USA).

### 4.8. Annexin V Analysis

Distribution of apoptotic cells was determined using the Muse^TM^ Annexin V & Dead Cell Kit (Luminex, Austin, TX, USA) according to the manufacturer’s instructions. Briefly, 1 × 10^5^ cells were seeded in 60 mm dishes and, after 48 h, exposed to the different stimuli in medium supplemented with 1% FBS for 24 h. Cells were then detached with PBS 1X/EDTA (5 mm), centrifuged (300× *g*, 5 min), resuspended in Muse^TM^ Annexin V & Dead Cell reagent, and analyzed with Muse^TM^ Cell Analyzer Software_V1.5.0.0 (Luminex, Austin, TX, USA) following the manufacturer’s instructions.

### 4.9. Real-Time PCR

Total RNA extraction and reverse transcription to cDNA (1 μg RNA) were performed using TriFast II (Euroclone, Milan, Italy, #EMR517100) and RevertAid RT kit (Thermo Fisher Scientific, Milan, Italy, #K1691), according to the manufacturer’s instructions. cDNAs were treated with DNA-free DNase (Invitrogen; Thermo Fisher Scientific, Milan, Italy, # 18068015) and reactions were performed with 50 ng cDNA, 100 nM of each primer and the Luna Universal qPCR master mix (New England BioLabs, Ipswich, MA, USA, #M3003E) using the ABI-Prism 7300 (Applied Biosystems; Thermo Fisher Scientific, Milan, Italy). The following primer pairs were used: *IGF1*, forward 5′-CTCTTCAGTTCGTGTGTGGAGAC-3′, reverse 5′-CAGCCTCCTTAGATCACAGCTC-3′ (XM_054371953.1); *CCND1*, forward 5′-ATGTGTGCAGAAGGAGGTCC-3′, reverse 5′-CCTTCATCTTAGAGGCCACG-3′ (NM_053056.3); *CCNE*; forward 5′-CAGATTGCAGAGCTGTTGGA-3′, reverse 5′-TCCCCGTCTCCCTTATAACC-3′ (NM_001238.4); *CCNB1*, forward 5′-CGAAGATCAACATGGCAGG-3′, reverse 5′-CTTGGAGAGGCAGTATCAACC-3′ (NM_001354845.2); *MYC*, forward 5′-AGCGACTCTGAGGAGGAACA-3′, reverse 5′-CTCTGACCTTTTGCCAGGAG-3′ (NM_002467.6); *COX2*, forward 5′-CGGTGAAACTCTGGCTAGACAG-3′, reverse 5′-GCAAACCGTAGATGCTCAGGGA-3′ (NM_000963.4); *ECAD*, forward 5′-GCCTCCTGAAAAGAGAGTGGAAG-3′, reverse 5′-TGGCAGTGTCTCTCCAAATCCG-3′ (NM_001317185.2); *NCAD*, forward 5′-CCTCCAGAGTTTACTGCCATGAC-3′, reverse 5′-GTAGGATCTCCGCCACTGATTC-3′ (NM_001308176.2); *VIM*, forward 5′-AGGCAAAGCAGGAGTCCACTGA-3′, reverse 5′-ATCTGGCGTTCCAGGGACTCAT-3′ (NM_003380.5); *MMP2*, forward 5′-ACCTGGATGCCGTCGTGGAC-3′, reverse 5′-TGTGGCAGCACCAGGGCAGC-3′ (NM_001302510.1); *MMP9*, forward 5′-TTGACAGCGACAAGAAGTG-3′, reverse 5′-GCCATTCACGTCGTCCTTAT-3′ (NM_004994.2); *VEGF*, forward 5′-ATCTTCAAGCCATCCTGTGTGC-3′, reverse 5′-CAAGGCCCACAGGGATTTTC-3′(NM_001287044.1); and *18S rRNA*, forward 5′-CCCATTCGAACGTCTGCCCTATC-3′, reverse 5′-TGCTGCCTTCCTTGGATGTGGTA-3′ (NR_146144.1) (designed with Primer 3 software, http://www.primer3.org/). *18S rRNA* was used as an endogenous control. Relative quantification was performed using the comparative Ct (2−ΔΔCt) method.

### 4.10. Western Blot Analysis

Protein extraction and Western blot analysis were performed as described previously [34]. Proteins (50 μg) were separated by SDS-PAGE. After blocking with 5% BSA in Tris-buffered saline with 0.1% Tween for 2 h at room temperature, membranes were incubated overnight at 4 °C with the specific antibody (dilution 1:1000 for GHRH-R, p-Akt, p-Stat3, p-NF-κB p65, p27, Bax, COX-2 and E-cadherin and 1:500 for p53, p21, c-Myc, and Bcl-2). Blots were reprobed with the respective total antibodies or actin for protein normalization (dilution, 1:1000 for NF-κB p65 and Akt and 1:500 for STAT3 and actin). Immunoreactive proteins were visualized using horseradish peroxidase-conjugated goat anti-mouse (RRID:AB_10015289), mouse anti-goat (RRID:AB_2339057), or goat anti-rabbit (RRID:AB_2307391) secondary antibodies (1:10,000, Jackson ImmunoResearch, West Grove, PA, USA) by enhanced chemiluminescence substrate (ECL, Bio-Rad, Milan, Italy, #170-5061) using ChemiDoc XRS (Bio-Rad, Milan, Italy); densitometric analysis was performed with Quantity One software (Bio-Rad, Milan, Italy). Original images are provided in Supplementary Figure S1.

### 4.11. Gelatin Zymography

Gelatinolytic activity of MMP9 and MMP2 in A549 conditioned medium was determined by gelatin zymography. Equal amounts of proteins (30 μg) were separated in SDS-PAGE under non-reducing conditions on 8% SDS-polyacrylamide gel co-polymerized with 4 mg/mL of gelatin. After electrophoresis, the gel was washed in 2.5% Triton X-100 solution for 1 h to remove SDS and then incubated in developing buffer (1% Triton X-100, 50 mM Tris-HCl, pH 7.5, 5 mM CaCl_2_, and 1 µM ZnCl_2_) at 37°C for 24 h, which allows substrate degradation. Finally, gel was stained with 0.1% Coomassie Brilliant Blue solution (Sigma-Aldrich; Merck, Milan, Italy) for 1 h and then destained in 50% methanol and 5% acetic acid. Proteolytic bands were visualized as a clear band against a blue background. MMP9 and MMP2 enzymatic activity were determined by quantification of clear bands using ChemiDoc XRS (Bio-Rad, Milan, Italy); densitometric analysis was performed with Quantity One software (Bio-Rad, Milan, Italy). Original images are provided in Supplementary Figure S1.

### 4.12. Statistical Analysis

Results are presented as means ± SEM. Significance was calculated by one-way ANOVA, followed by Dunnett’s or Tukey’s multiple comparison test for post hoc analysis. Analysis was performed using GraphPad Prism 8.0 (San Diego, CA, USA; RRID:SCR_002798). Significance was established at *p* < 0.05. Combination index (CI) values were calculated with CompuSyn software (www.combosyn.com; RRID:SCR-022931).

## Data Availability

The data used in the current study are available from the corresponding author upon reasonable request.

## Supplementary Materials

The following supporting information can be downloaded at: https://www.mdpi.com/article/10.3390/ijms26073267/s1.
