# Supplementary material for: Growth Hormone-Releasing Hormone Antagonists Increase Radiosensitivity in Non-Small Cell Lung Cancer Cells
Source: Int J Mol Sci. 2025 Apr 1;26(7):3267. doi: 10.3390/ijms26073267 (PMC11990011; doi:10.3390/ijms26073267)
Supplement: Supplementary file 1 [file ijms-26-03267-s001.zip › Figure Caption.docx]

**Figure S1:** Original Western blot images
