# Supplementary material for: Growth Hormone-Releasing Hormone Antagonists Increase Radiosensitivity in Non-Small Cell Lung Cancer Cells
Source: Int J Mol Sci. 2025 Apr 1;26(7):3267. doi: 10.3390/ijms26073267 (PMC11990011; doi:10.3390/ijms26073267)

Gesmundo et al. ijms-3352732

Growth hormone-releasing hormone antagonists increase radiosensitivity in non-small cell lung cancer cells

Original Western blots and gelatin zimography

GHRH-R and SV1 expression in A549 and H522 cells (Figure 1A)

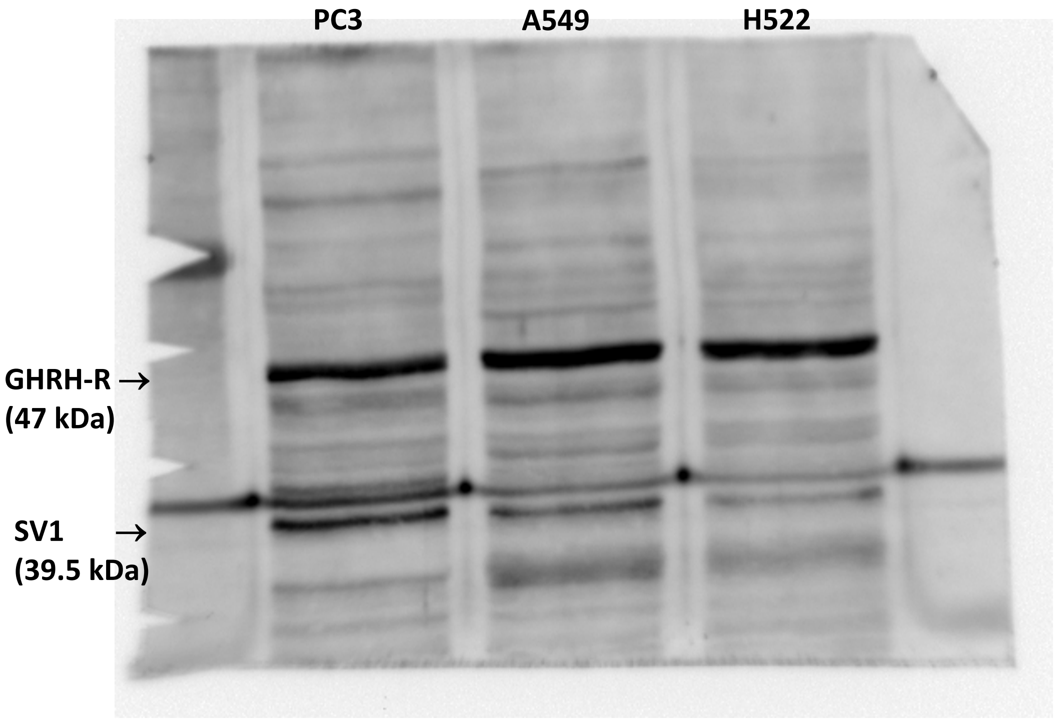

Actin for GHRH-R and SV1 expression in A549 and H522 cells (Figure 1A)

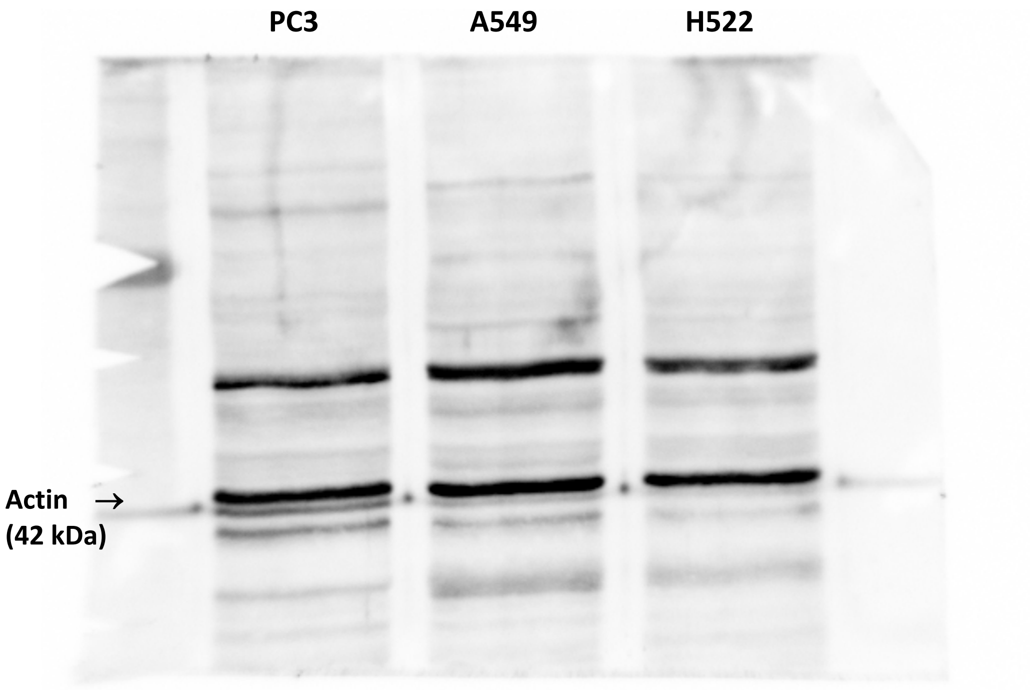

GHRH-R and SV1 expression in A549 cells exposed to M-690 and IR (Figure 3A)

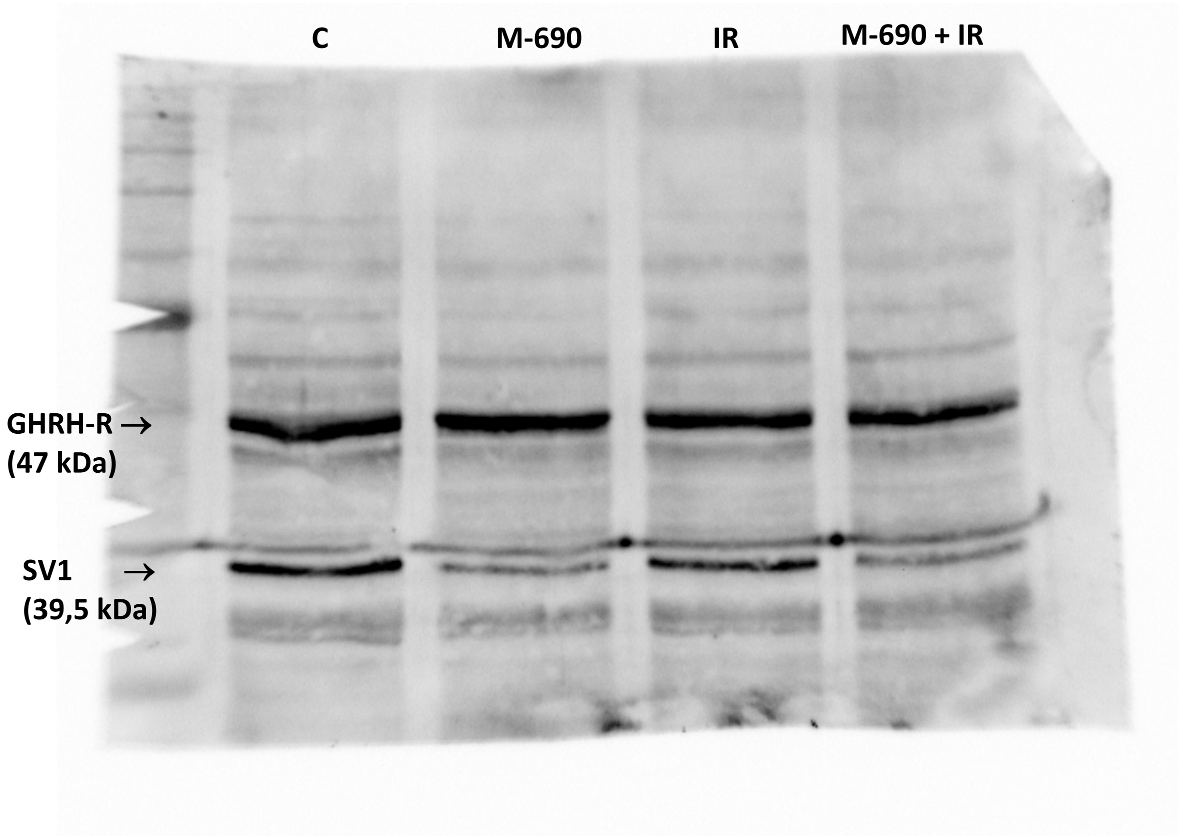

Actin for GHRH-R and SV1 expression in A549 cells (Figure 3A)

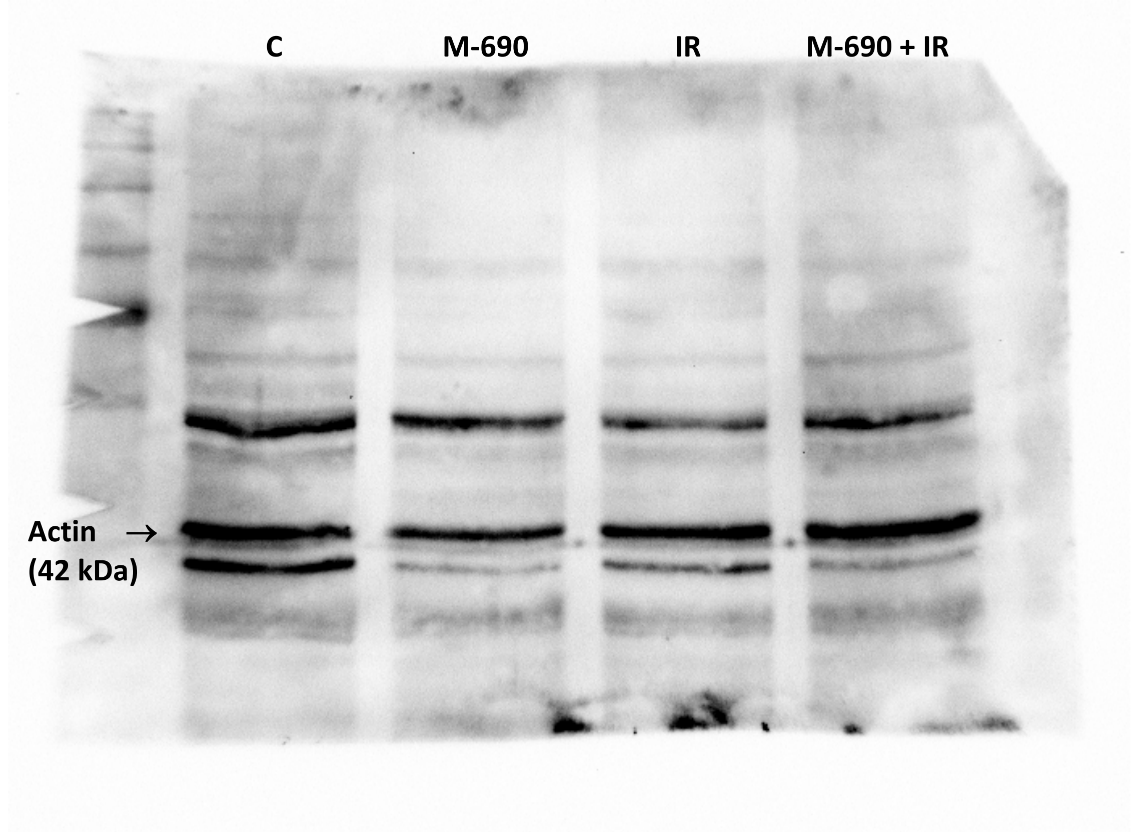

p21 in A549 cells exposed to M-690 and IR (Figure 4B)

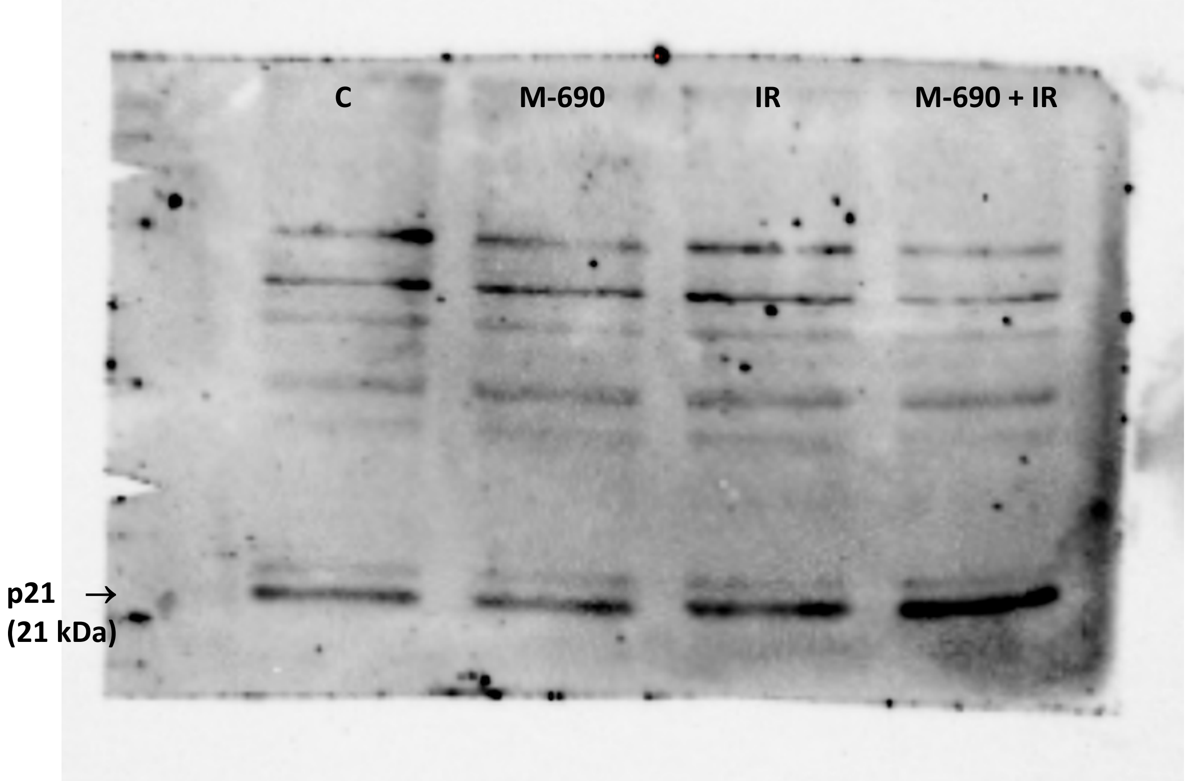

Actin for p21 in A549 cells (Figure 4B)

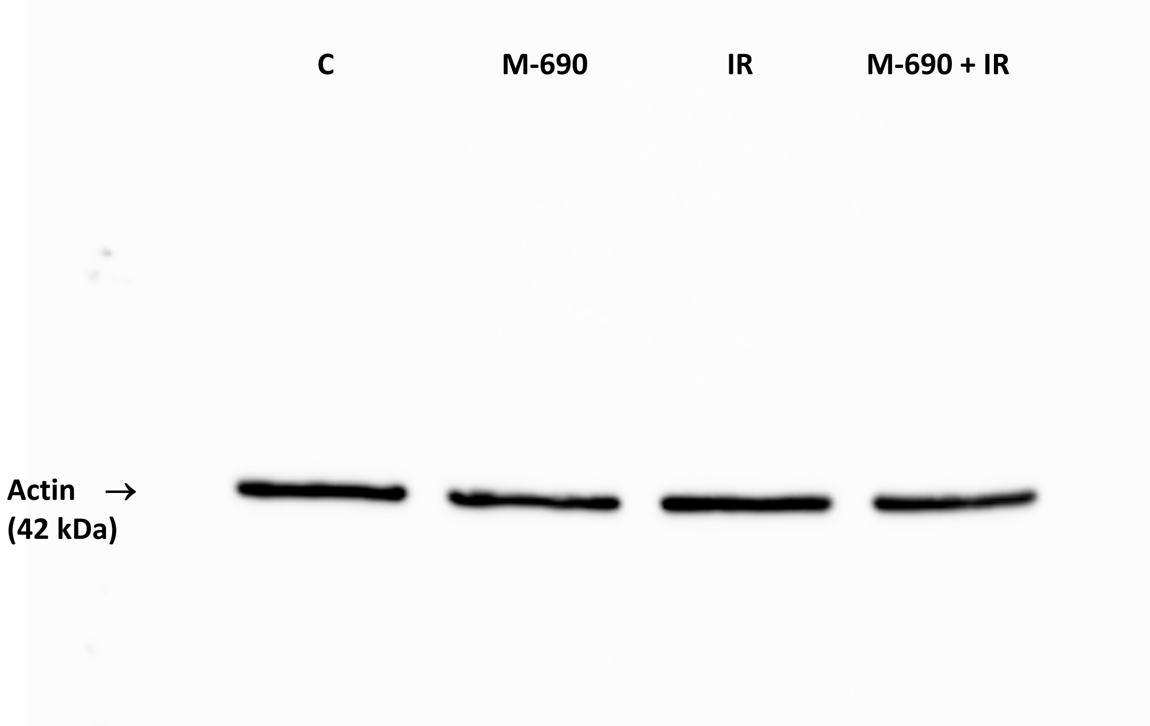

p27 in A549 cells exposed to M-690 and IR (Figure 4C)

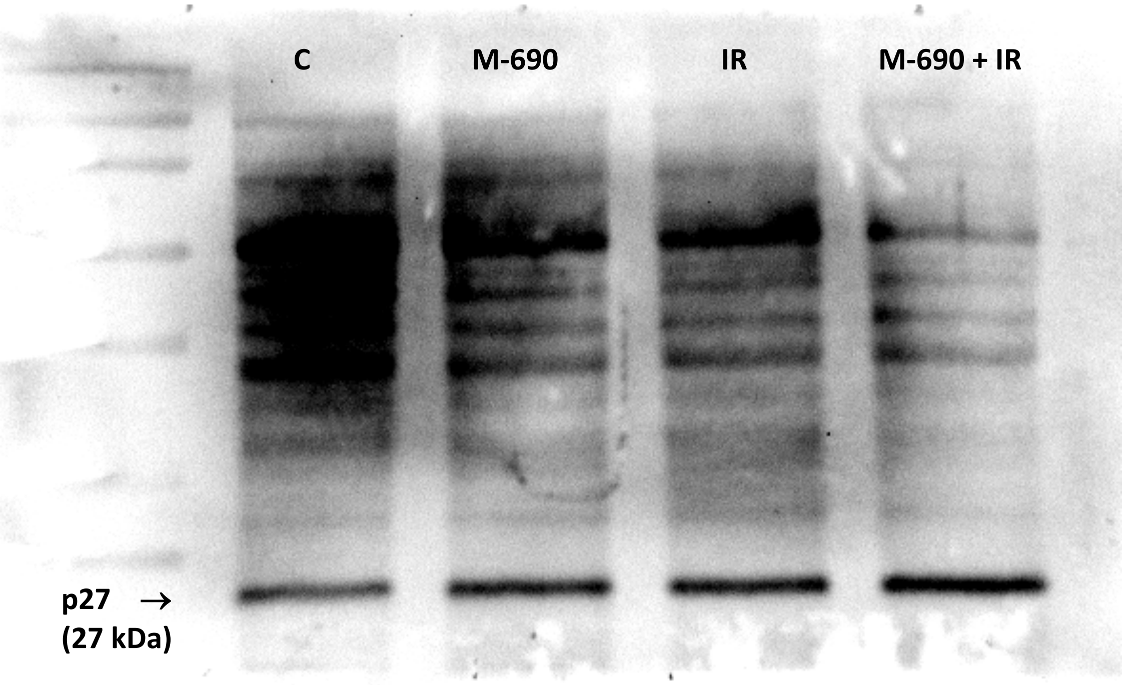

Actin for p27 in A549 cells (Figure 4C)

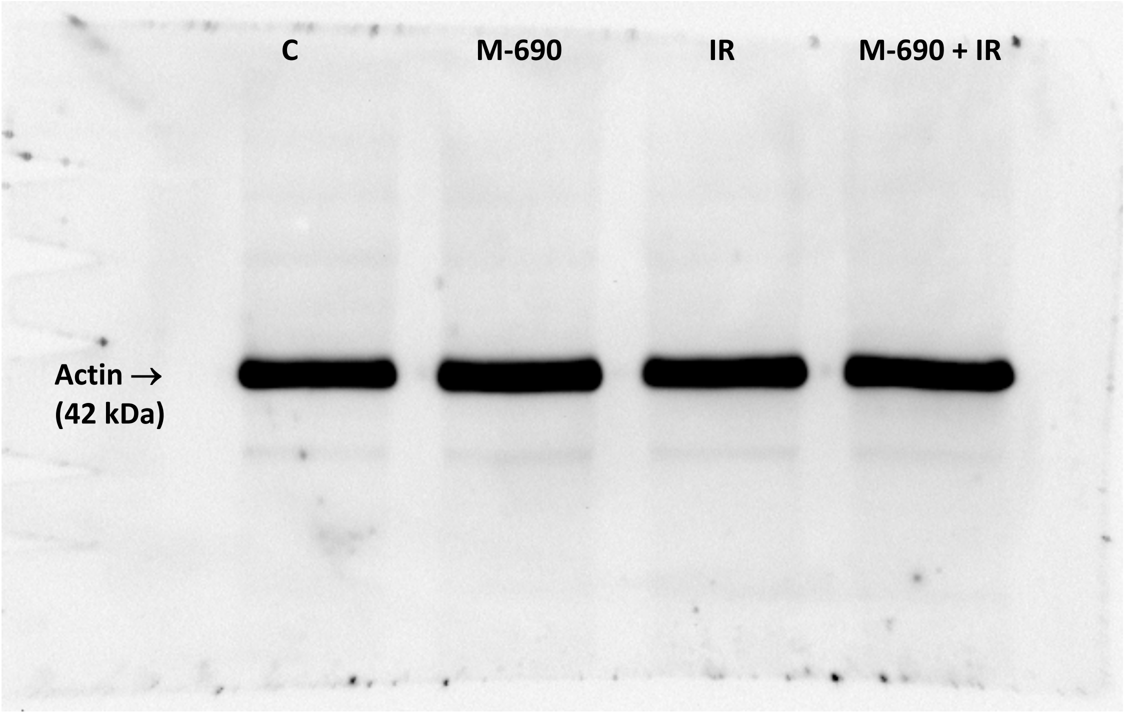

c-Myc in A549 cells exposed to M-690 and IR (Figure 4J)

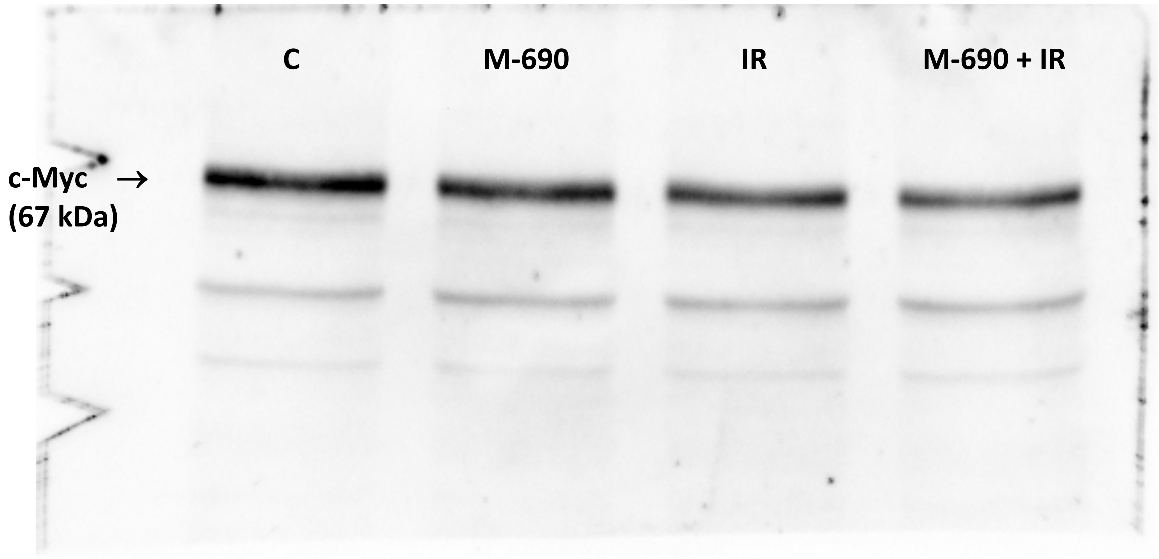

Actin for c-Myc in A549 cells (Figure 4J)

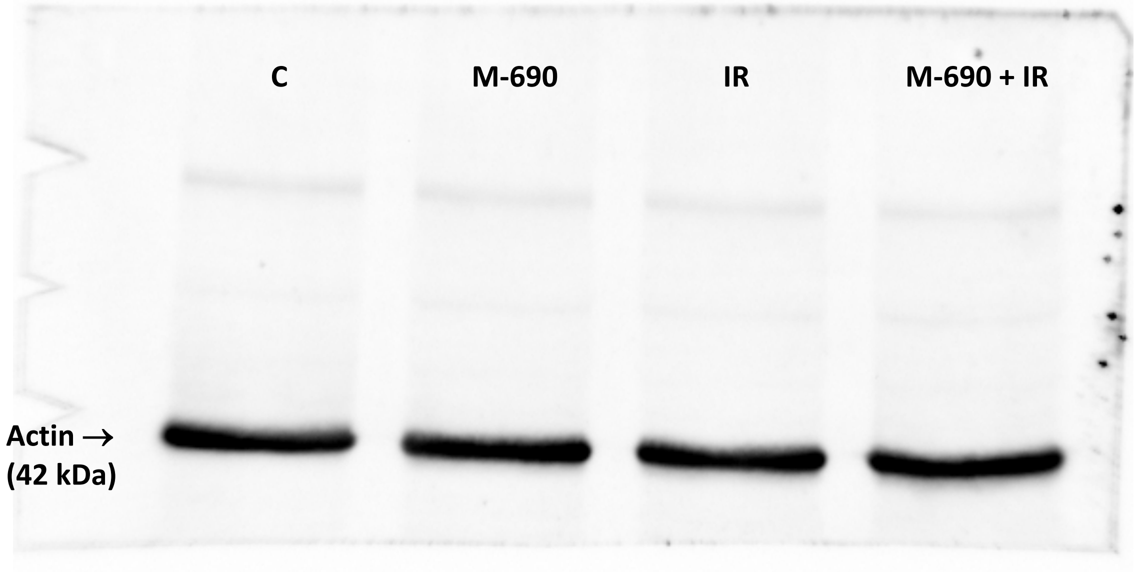

p53 in A549 cells exposed to M-690 and IR (Figure 5C)

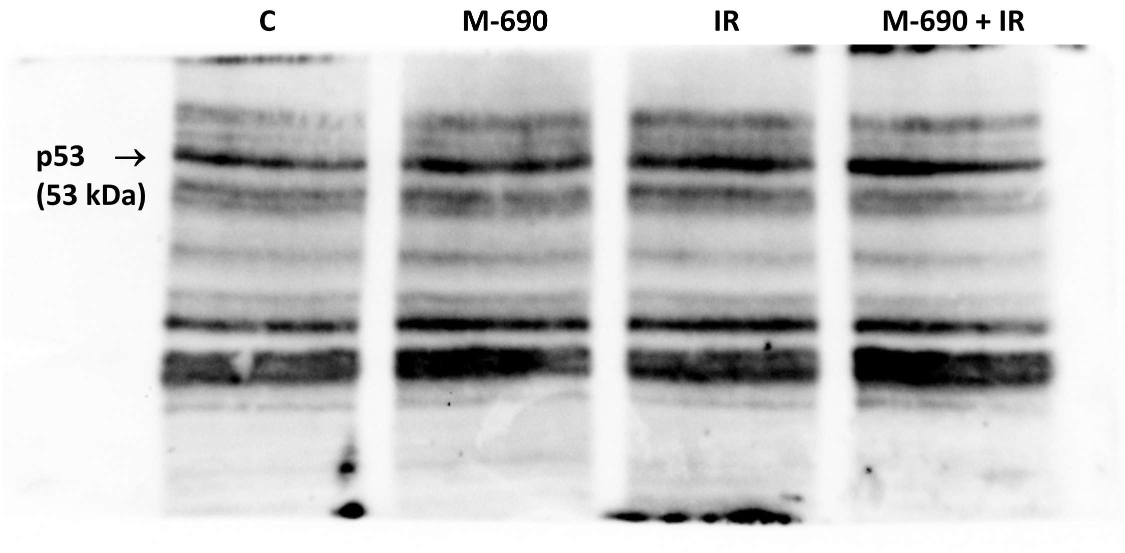

Actin for p53 in A549 cells (Figure 5C)

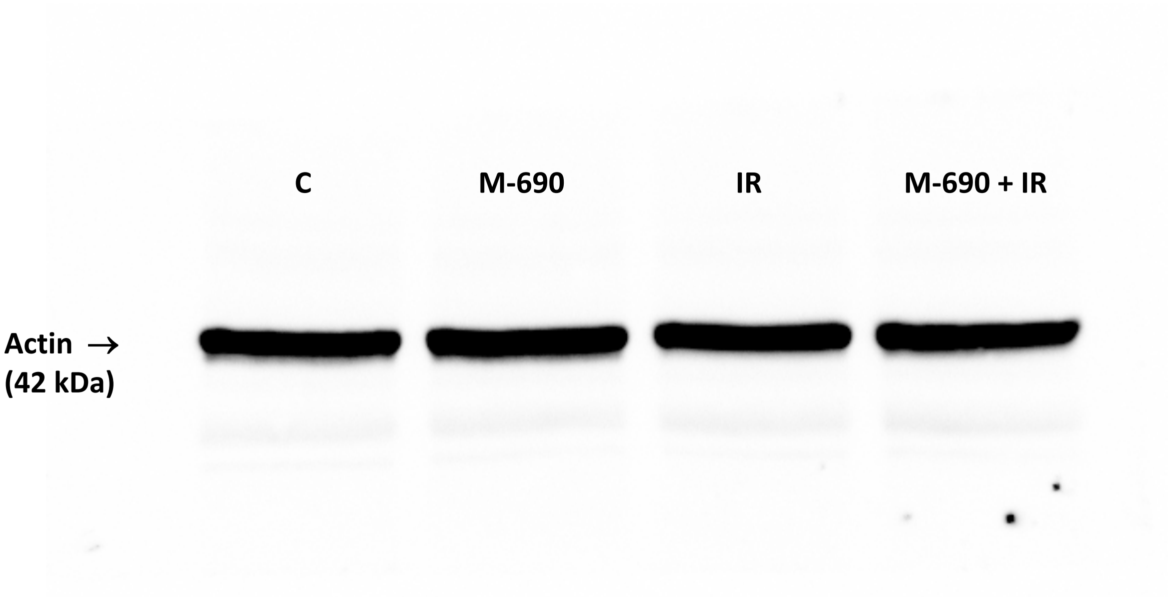

Bax in A549 cells exposed to M-690 and IR (Figure 5E)

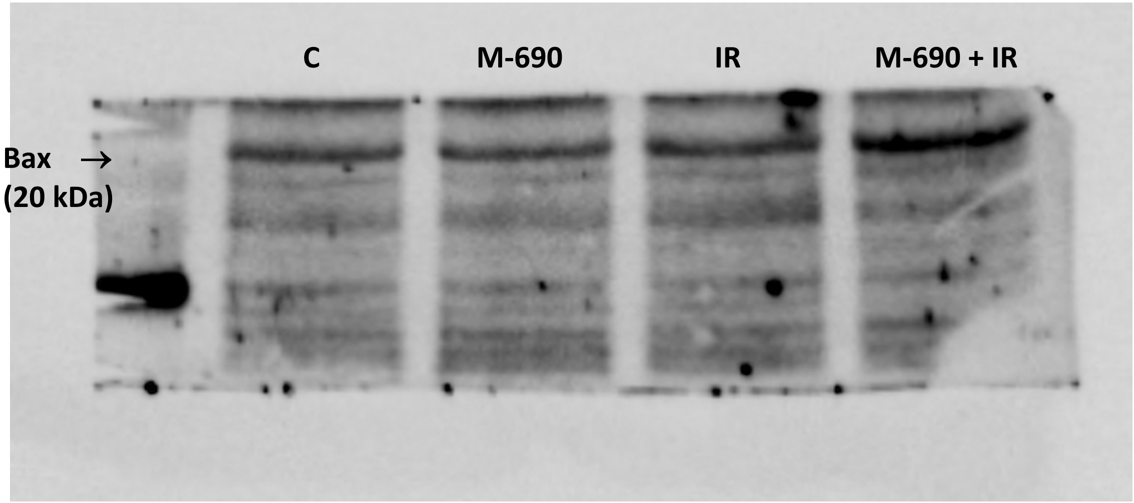

Actin for Bax in A549 cells (Figure 5E)

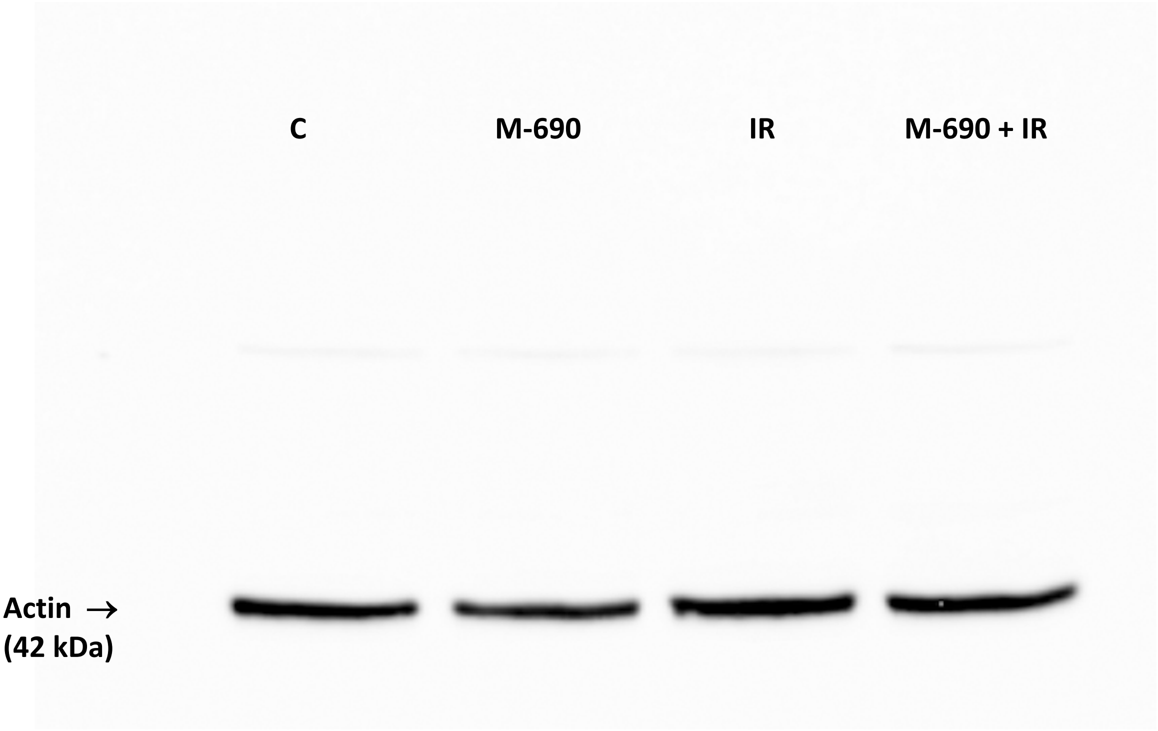

Bcl-2 in A549 cells exposed to M-690 and IR (Figure 5F)

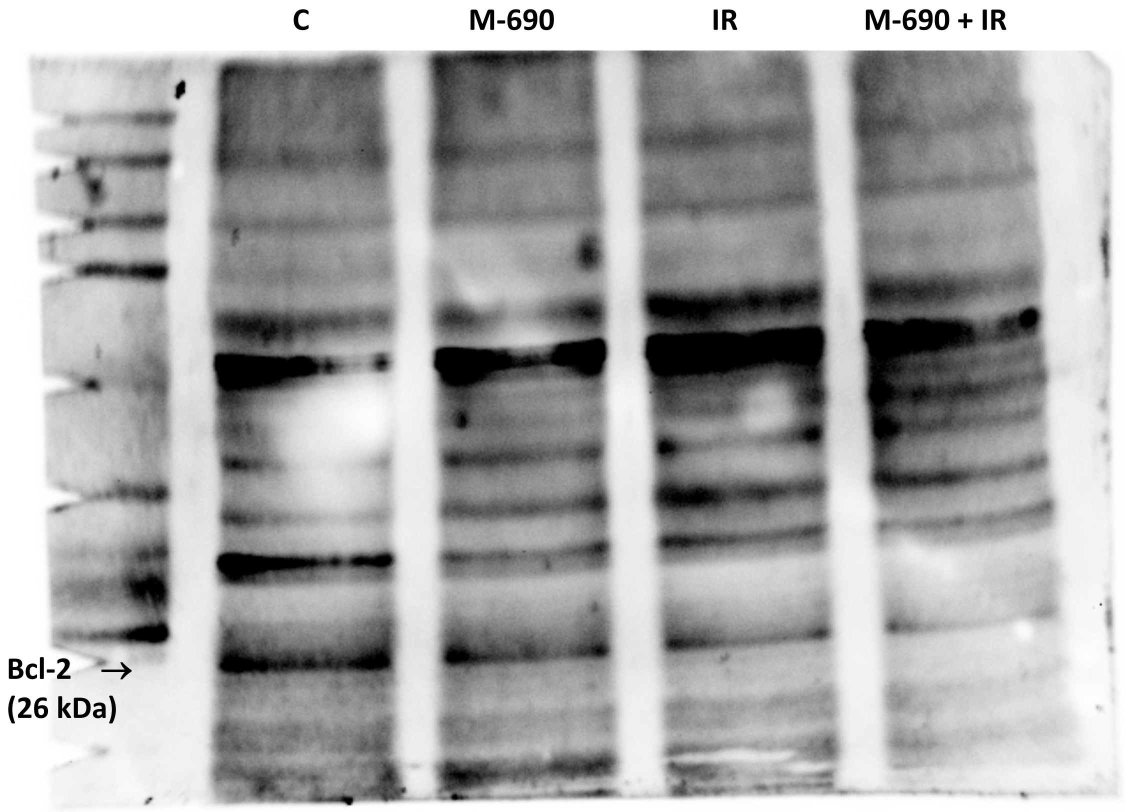

Actin for Bcl-2 in A549 cells (Figure 5F)

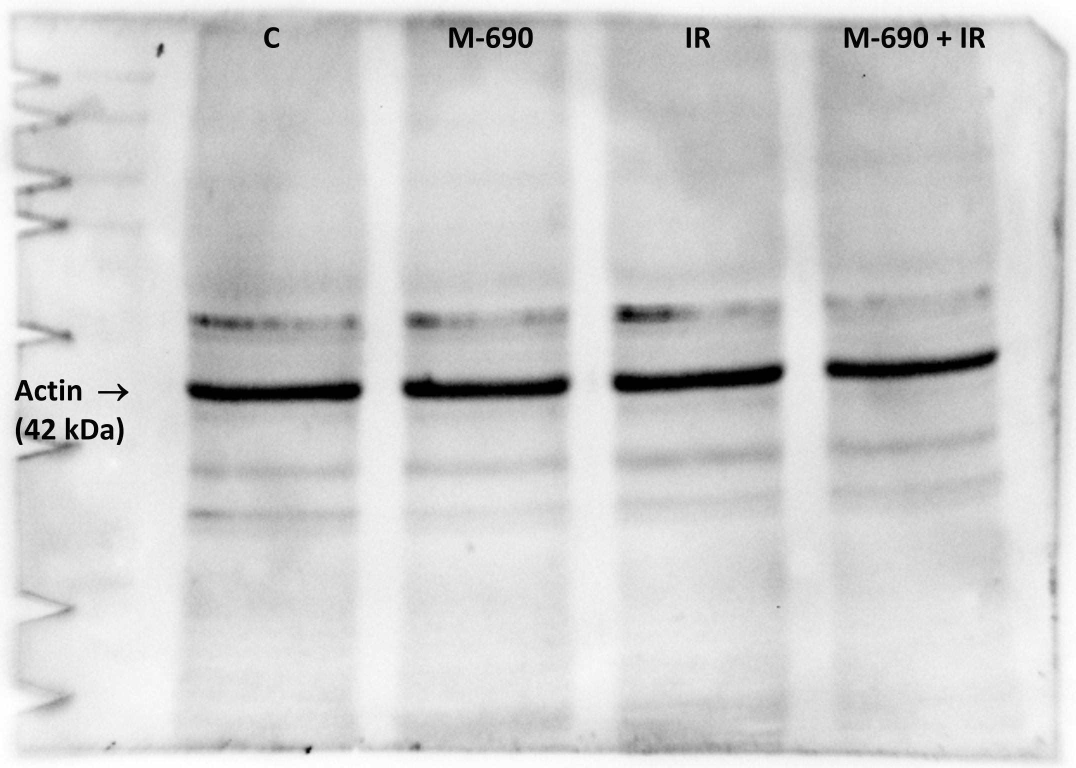

p-Akt in A549 cells exposed to M-690 and IR (Figure 6A)

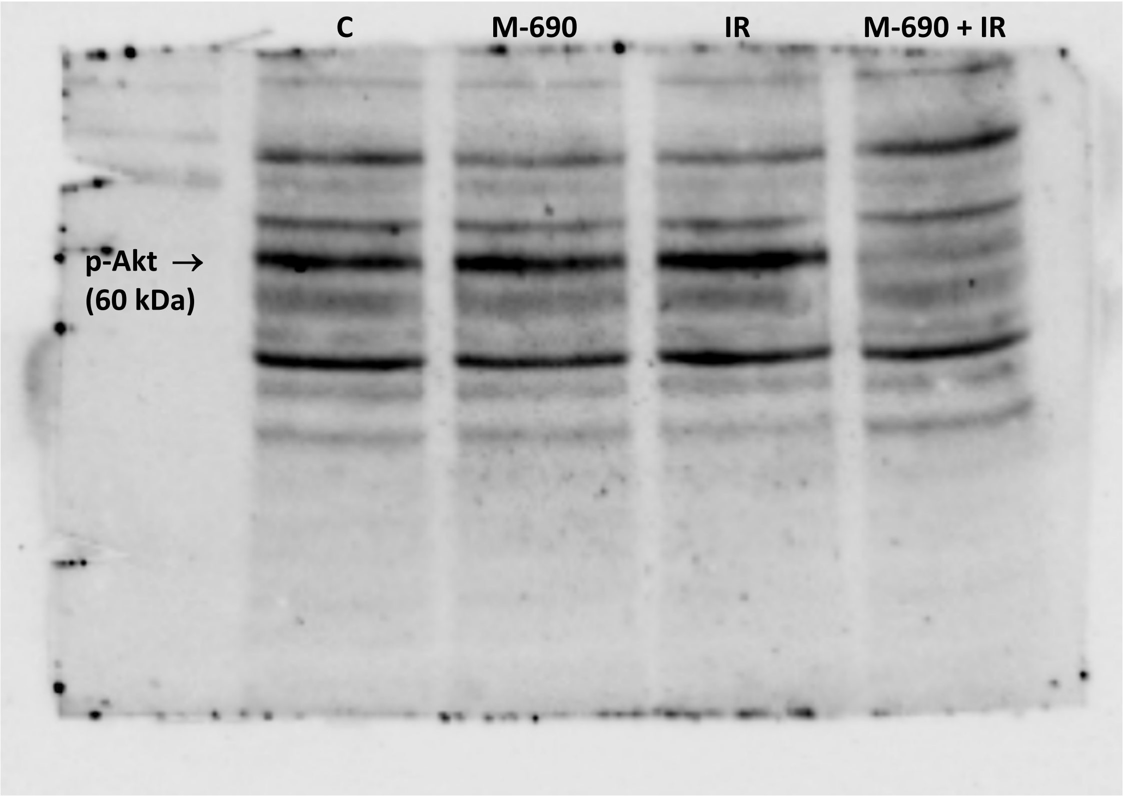

Akt in A549 cells (Figure 6A)

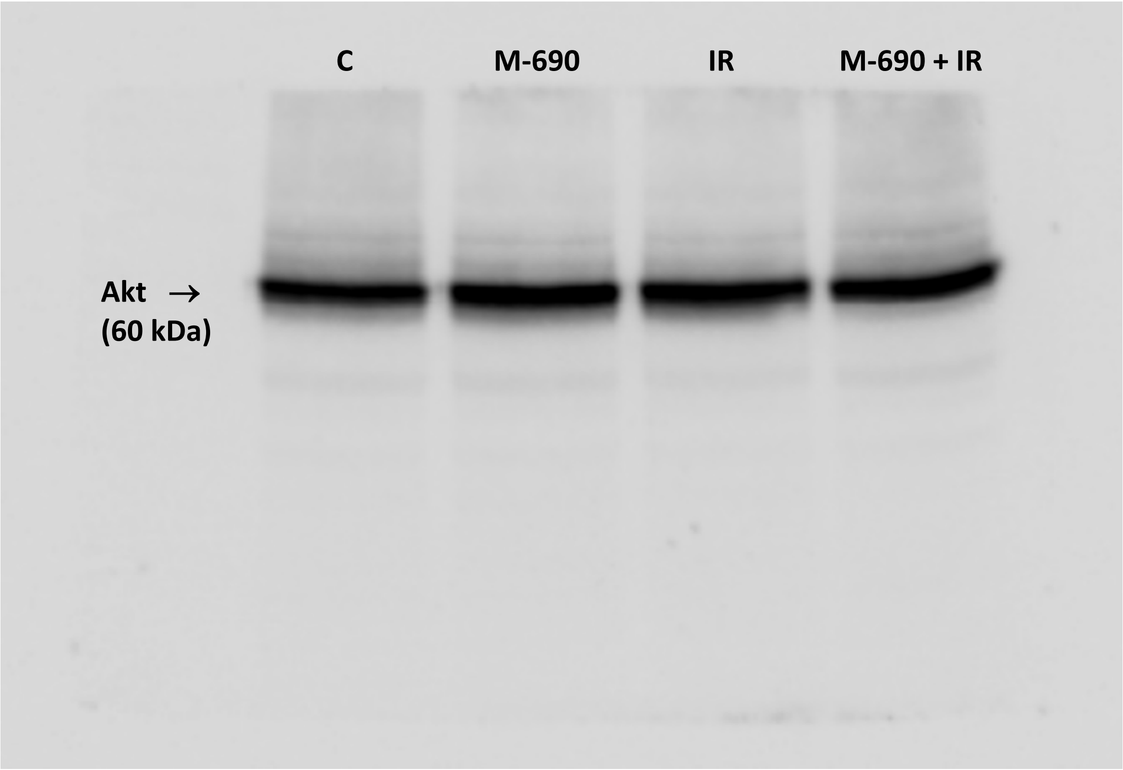

p-STAT3 in A549 cells exposed to M-690 and IR (Figure 6B)

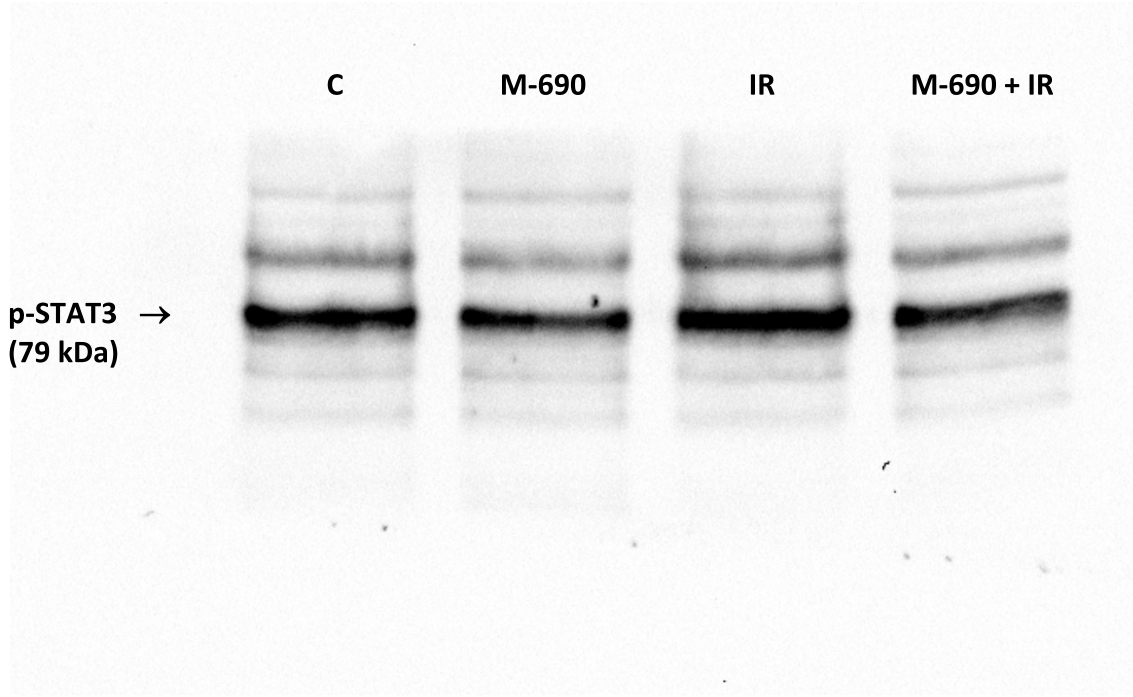

STAT3 in A549 cells (Figure 6B)

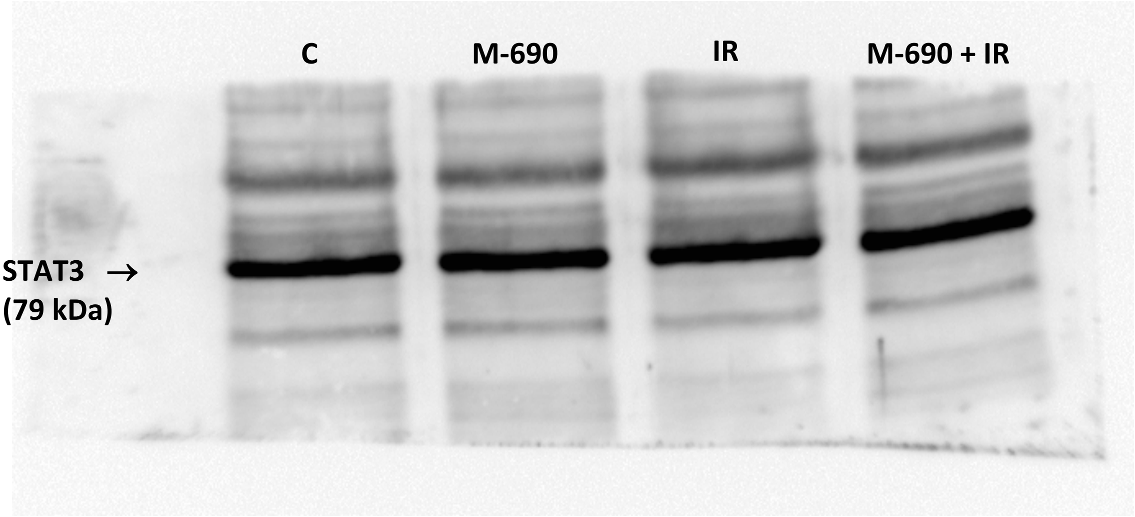

p-p65 in A549 cells exposed to M-690 and IR (Figure 6C)

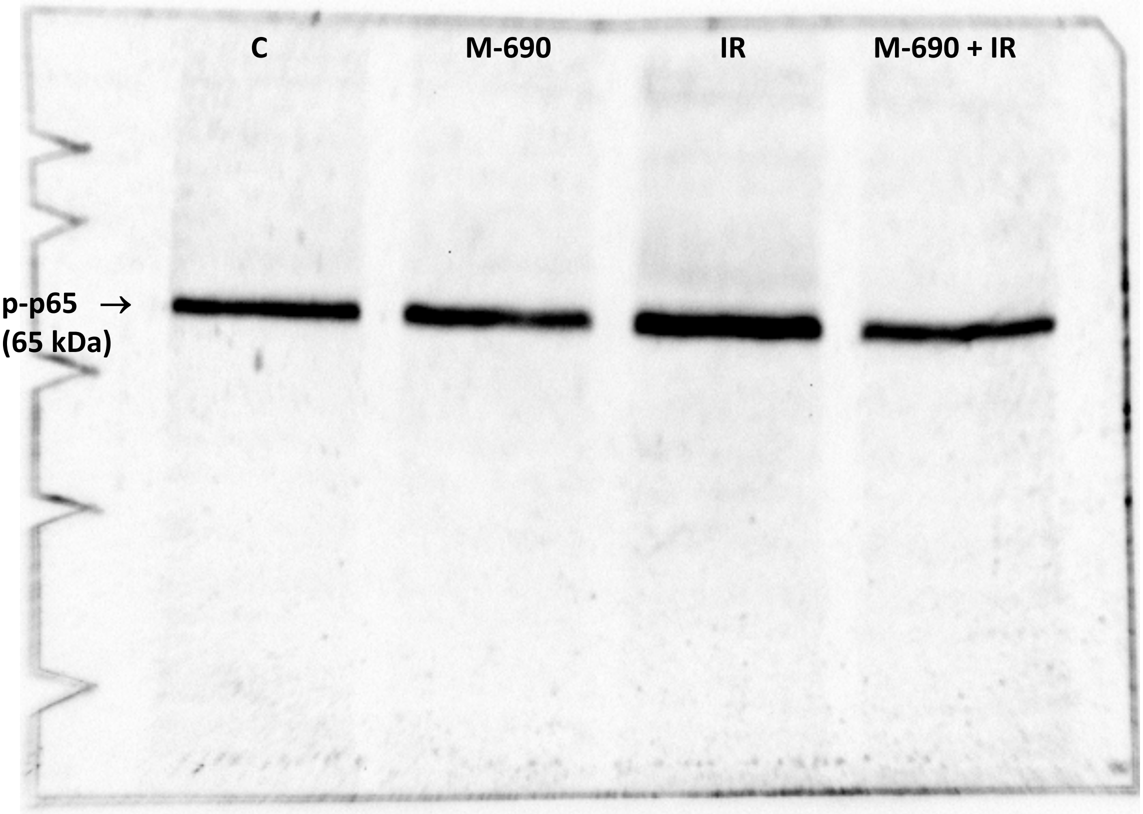

p65 in A549 cells (Figure 6C)

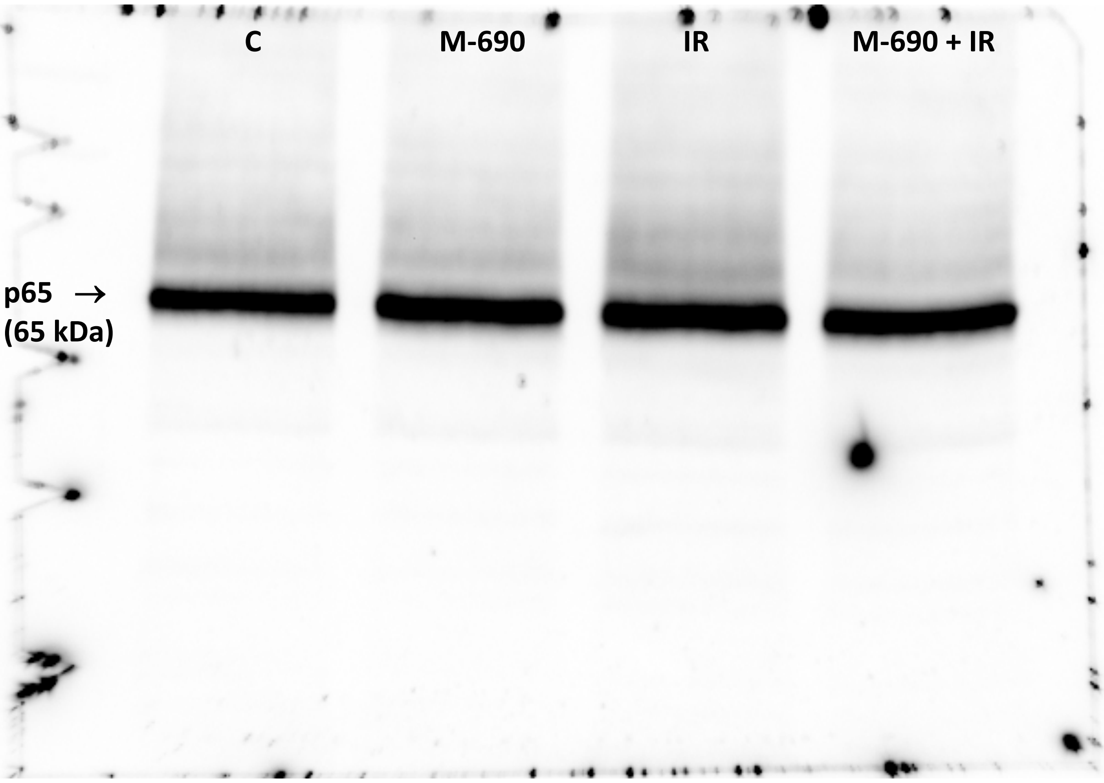

COX-2 in A549 cells exposed to M-690 and IR (Figure 6E)

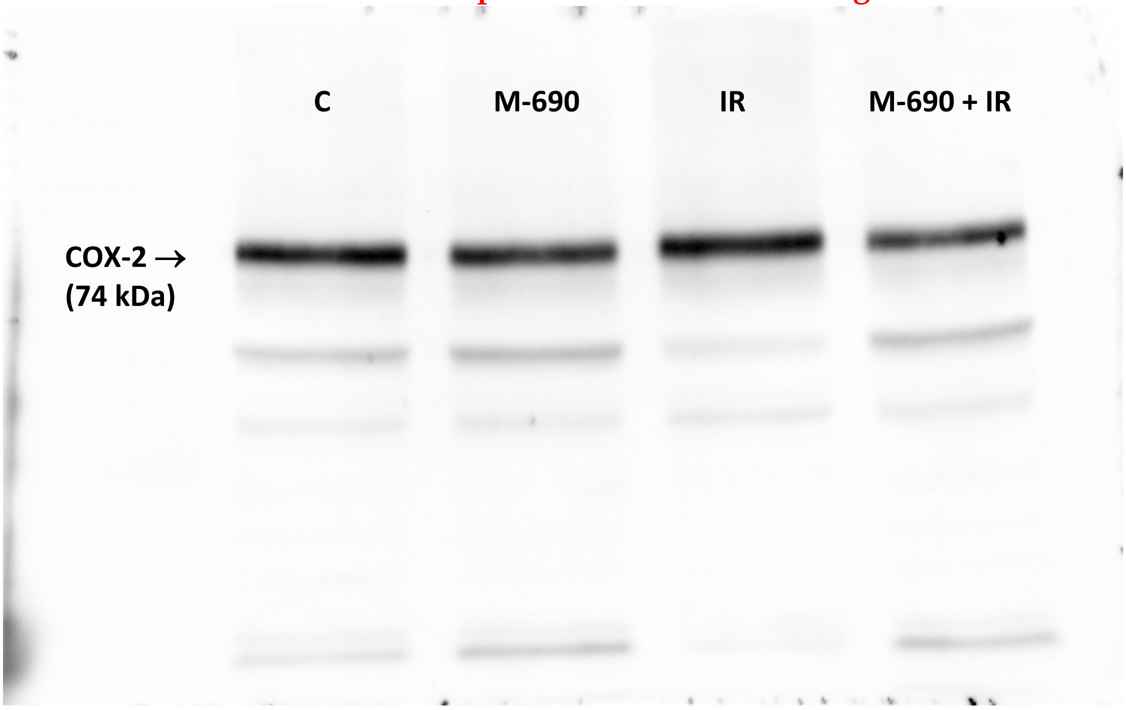

Actin for COX-2 in A549 cells (Figure 6E)

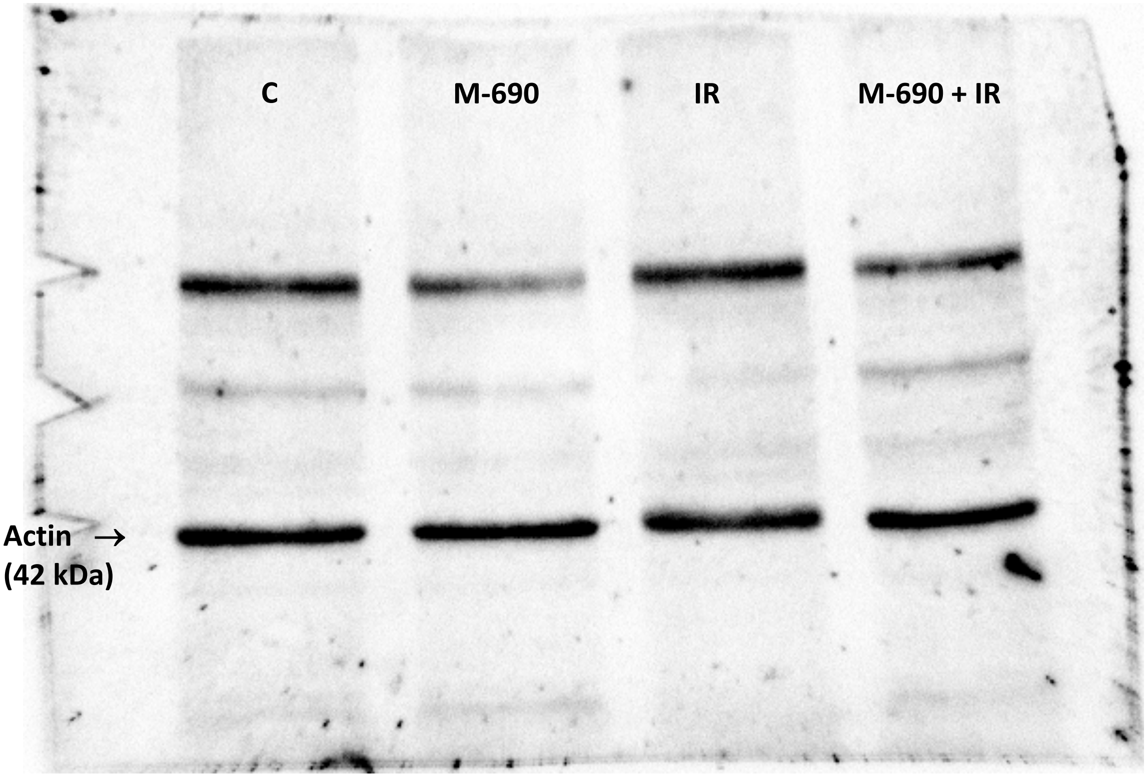

E-cadherin in A549 cells exposed to M-690 and IR (Figure 6G)

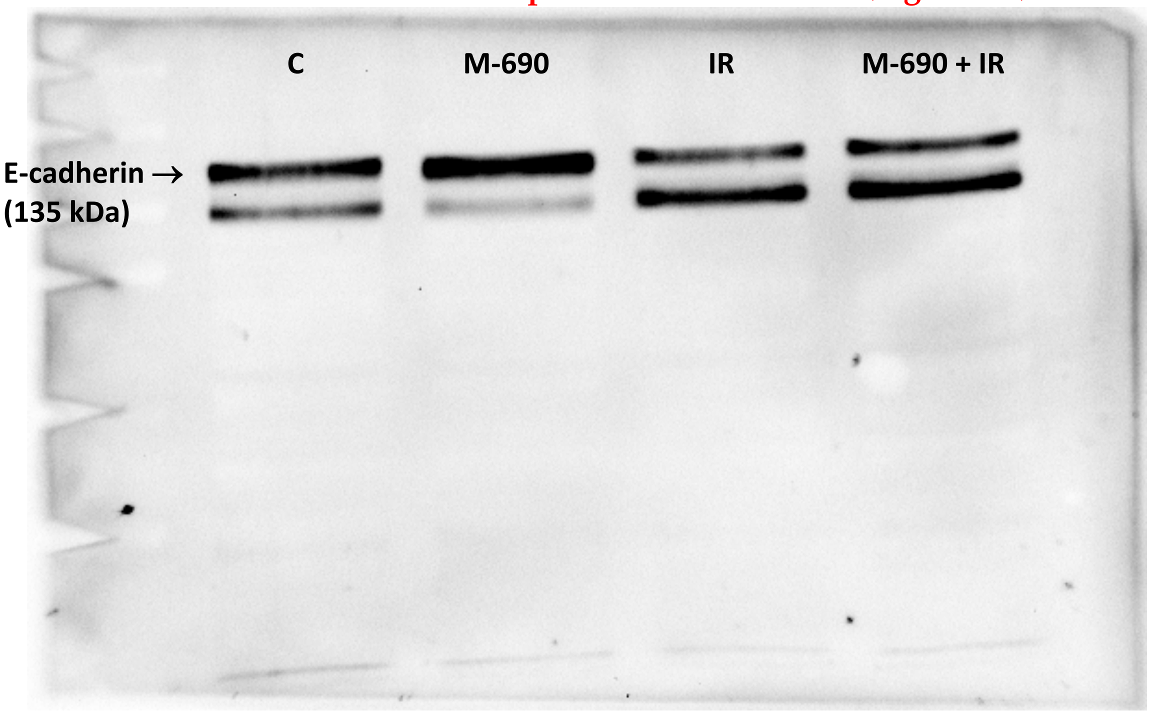

Actin for E-cadherin in A549 cells (Figure 6G)

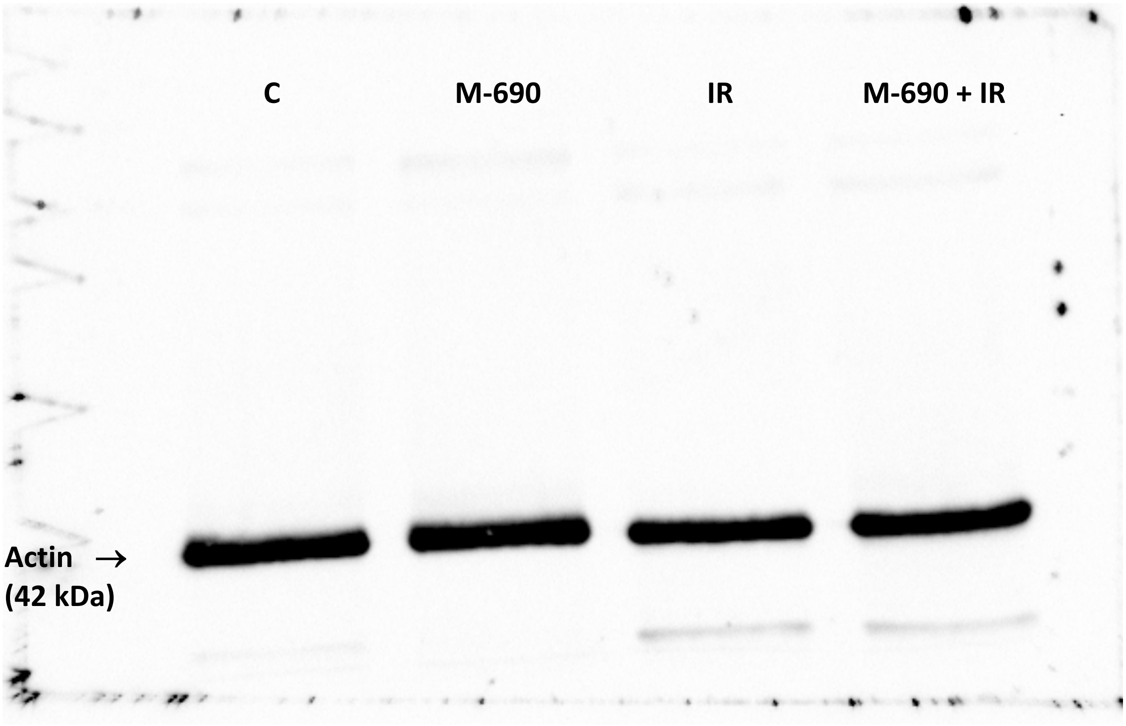

Gelatin zymography for MMP-9 and MMP-2 activity in A549 cells (Figure 6J)

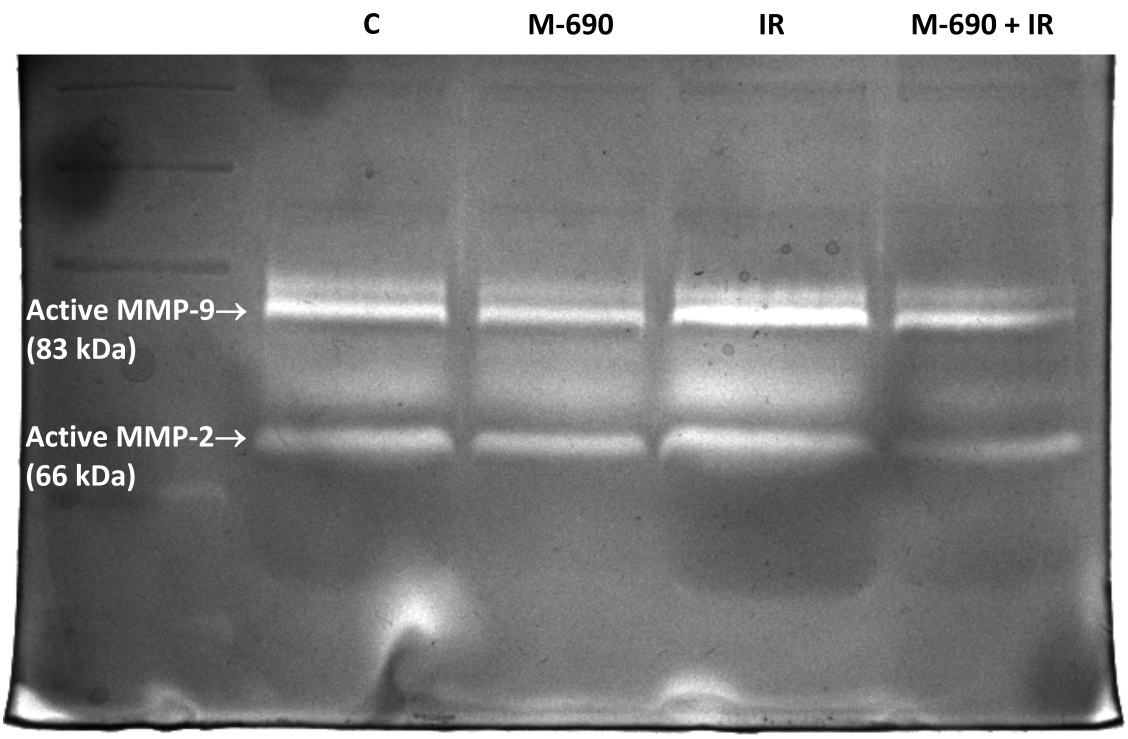

Supplement: Supplementary file 1 [file ijms-26-03267-s001.zip › Figure S1.pdf]
